# Harnessing protein sensing ability of electrochemical biosensors via a controlled peptide receptor–electrode interface

**DOI:** 10.1186/s12951-023-01843-0

**Published:** 2023-03-21

**Authors:** Ji Hong Kim, Jae Hwan Shin, Bumjun Park, Chae Hwan Cho, Yun Suk Huh, Chang-Hyung Choi, Jong Pil Park

**Affiliations:** 1 Basic Research Laboratory, Department of Food Science and Technology, Chung-Ang University, Anseong, 17546 Republic of Korea; 2 NanoBio High-Tech Materials Research Center, Department of Biological Sciences and Bioengineering, Inha University, 100 Inha-Ro, Incheon, 22212 Republic of Korea; 3 School of Chemical Engineering, Yeungnam University, 280 Daehak-ro, Gyeongsan, Gyeongbuk 38541 Republic of Korea

**Keywords:** Cathepsin B, Biomarker, Affinity peptide, Electrochemistry, Diagnosis

## Abstract

**Background:**

Cathepsin B, a cysteine protease, is considered a potential biomarker for early diagnosis of cancer and inflammatory bowel diseases. Therefore, more feasible and effective diagnostic method may be beneficial for monitoring of cancer or related diseases.

**Results:**

A phage-display library was biopanned against biotinylated cathepsin B to identify a high-affinity peptide with the sequence WDMWPSMDWKAE. The identified peptide-displaying phage clones and phage-free synthetic peptides were characterized using enzyme-linked immunosorbent assays (ELISAs) and electrochemical analyses (impedance spectroscopy, cyclic voltammetry, and square wave voltammetry). Feasibilities of phage-on-a-sensor, peptide-on-a-sensor, and peptide-on-a-AuNPs/MXene sensor were evaluated. The limit of detection and binding affinity values of the peptide-on-a-AuNPs/MXene sensor interface were two to four times lower than those of the two other sensors, indicating that the peptide-on-a-AuNPs/MXene sensor is more specific for cathepsin B (good recovery (86–102%) and %RSD (< 11%) with clinical samples, and can distinguish different stages of Crohn’s disease. Furthermore, the concentration of cathepsin B measured by our sensor showed a good correlation with those estimated by the commercially available ELISA kit.

**Conclusion:**

In summary, screening and rational design of high-affinity peptides specific to cathepsin B for developing peptide-based electrochemical biosensors is reported for the first time. This study could promote the development of alternative antibody-free detection methods for clinical assays to test inflammatory bowel disease and other diseases.

**Supplementary Information:**

The online version contains supplementary material available at 10.1186/s12951-023-01843-0.

## Introduction

Cathepsins have attracted immense clinical attention as potential biomarkers for the early diagnosis of cancer [1–3]. It is the multifunctional enzymes that involve the whole pathogenesis and oncogenic process including differentiation and transition from cancer growth to metastasis. To date, more than 12 cathepsin families (from A to Z) have been found in organisms and rigorously characterized [4]. Among various cathepsin derivatives, cathepsin B (CTSB) is considered a potential biomarker for several types of cancers, such as prostate, pancreatic, colon, and breast cancers, as well as for osteoporosis, rheumatoid arthritis, and infectious diseases, and there is good correlation between tumor progression and the CTSB expression [5–8]. It is known that cathepsin B is a cysteine protease and can be involved in extracellular matrix (ECM) component degradation, cell–cell communication disruption and protease inhibitor expression [9, 10]. According to recent studies, CTSB levels in human fluids (serum or plasma) can be measured and remain within the diagnostic window after the onset of the inflammatory bowel diseases (IBD) including Crohn’s disease and Ulcerative colitis, enabling a non-invasive early detection of these diseases [11, 12]. For this and other purpose, chemical inhibitor and antibodies have been studied to suppress proteolytic activity of proteases in order to impose metastatic infiltration mediated by protease. Some of compound as inhibitor of cathepsin B have been identified in various resources, for example, microorganisms and natural products [13]. It was observed that the suppression of cathepsin B has high therapeutic significance, however, no direct targeting of cathepsin B with alternative binder and/or inhibitor over conventional reagents including antibodies or chemical inhibitor was reported.

The common features of the current antibody-based immunoassay and polymerase chain reaction (PCR) are considered as gold standard detection regime for detection of cancer biomarkers. However, these techniques have some bottlenecks in terms of operating cost, multiple sample preparation and labor-intensive and more effective biosensors have been developed [2, 3, 11, 12].

Phage display is a promising technology for the identification of potential peptide candidates with high affinity and specificity toward various target molecules (organic and inorganic materials) as well as for the development of therapeutic drugs and biosensing applications [14–16]. In phage display, M13 bacteriophages are genetically modified to expose small (generally, 12-mer, or 7-mer) unique peptides that are fused with the minor coat protein pIII (approximately five copies per phage) or major coat protein pVIII (approximately 2700 copies per phage) on their surface randomly, during biopanning [17]. One of the most interesting features of this technique is its ability to obtain large diversified peptide libraries on the phage surfaces in a short time [15, 17–20]. Interestingly, affinity peptides obtained by phage display are relatively smaller than the full-sized antibodies and thus can be cost-effectively mass produced. Further, peptides can be genetically manipulated to form a series of peptide derivatives are easily and reliably conjugated with other motifs, and can be immobilized on various surfaces for biosensor applications.

Electrochemical-based biosensors have attracted among the various analytical techniques because of their high sensitivity, selectivity, portability and diversity of target molecules, for example, drug, virus, and protein [17, 21, 22]. Previously, several advanced electrochemical sensors have been developed by combining with enzymes and nanomaterials, such as layered double hydroxides (LDHs) which is a two-dimensional anionic lamellar clay material with charge compensating anions and solvation molecules for detection of small molecules [23–25] and carbon-based nanocomposites (specifically, MXenes which are two-dimensional transitional metal compounds) [26–30]. In a recent study, Au nanoparticles (AuNPs) and methylene blue were incorporated as signal amplifiers on MXene nanocomposites to fabricate electrochemical biosensors for prostate-specific antigen (PSA) detection [31]. These sensors detected PSA with a limit of detection (LOD) of 0.83 pg/mL and a linear dynamic range of < 10 pico level. MXene-based nanocomposites have recently emerged as viable candidates for electrochemical sensors owing to their favorable physical and chemical properties including high electrical conductivities, biocompatibility, large surface areas, and a facile sensor layer and electrode functionalization [32]. As these common features of detection techniques are further developing combination with functional carbon nanomaterial and unique affinity peptide may be opened the widow as replacement over antibody-based immunoassay in biosensor as well as providing the basis for monitoring progression of cancer.

**Fig. 1:**
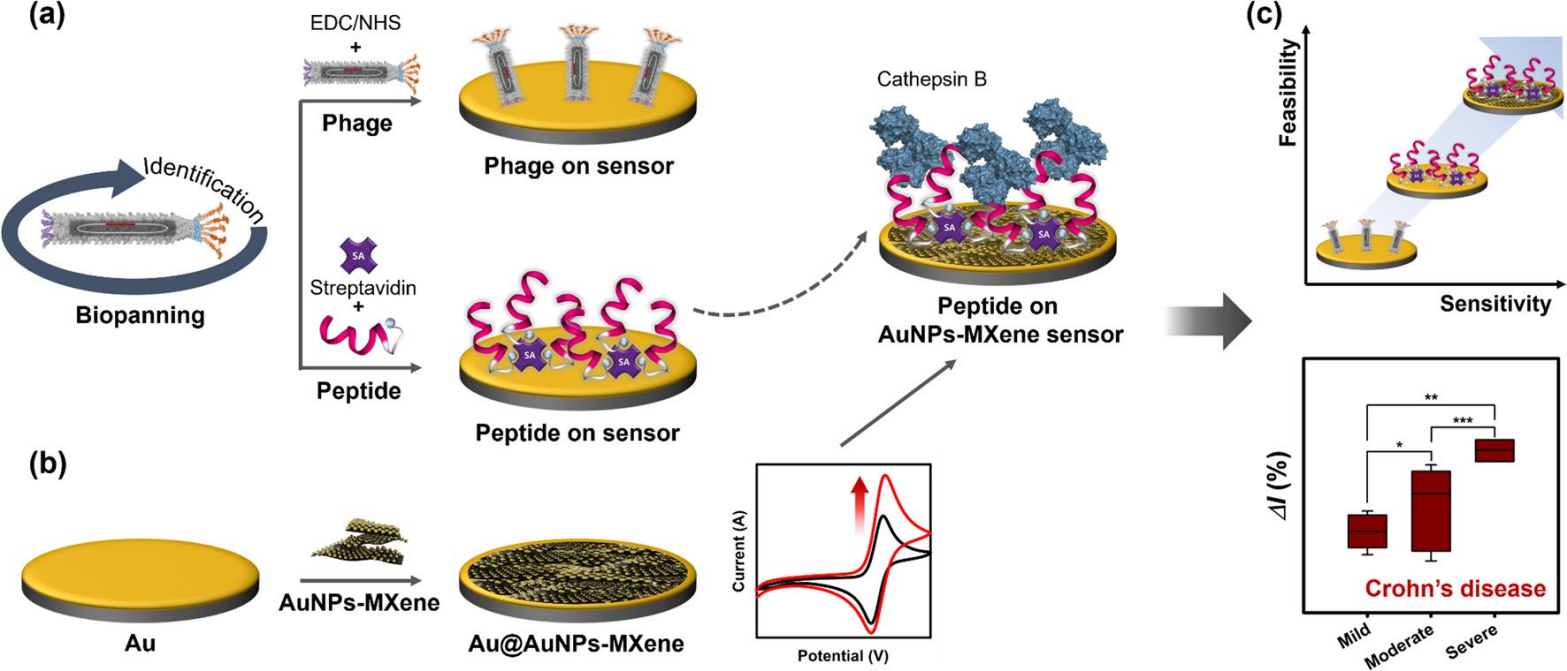
Schematic work flow of the affinity-based electrochemcial sensor used for the detection of cathepsin B. **a** 1. Phage-display selection, 2. Peptide-displayed whole phage immobilization on bare Au electrode using the MUA-EDC/NHS coupling, 3. Biotin labelled peptide synthesis and synthetic peptide immobilization on bare Au electrode using MUA-EDC/NHS coupling and streptavidin, **b** 1. AuNP-embedded MXene nanocomposite fabricated on Au electrode for high electrical conductivity, 2. Affinity peptide immobilization on AuNPs–MXene deposited electrode using MUA-EDC/NHS coupling and streptavidin, **c** Performances of developed sensors for target detection and validation in plama/serum from Crohn’s patients

This paper is the first ever report on the screening and rational design of high-affinity peptides for fabrication as well as the use of peptide-based electrochemical sensors for the accurate detection of cathepsin B. In this study, the M13 phage peptide library was biopanned to screen for high-affinity peptides for cathepsin B. The relative binding affinities of the phage clones and synthetic peptides were investigated by enzyme-linked immunosorbent assays (ELISAs) and electrochemical analyses including electrochemical impedance spectroscopy (EIS), cyclic voltammetry (CV) and square wave voltammetry (SWV). In addition, the sensor electrode was modified with a combination of AuNPs and a MXene material to improve the sensor performance (Fig. 1). This general approach could be extended to develop new type of biosensor for the various target molecules.

## Materials and methods

### Chemicals

Cathepsin B proteins, purified from human liver (~ 27.5 kDa, > 95% purity), were purchased from ENZO (NY, USA). The compounds 2,2ʹ-azino-bis(3-ethylbenzothiazoline-6-sulfonic acid (ABTS), 11-mercaptoundecanoic acid (MUA), *N*-(3-dimethylaminopropyl)-*N*′-ethylcarbodiimide hydrochloride (EDC), Tween 20, *N*-hydroxysuccinimide (NHS), potassium ferricyanide (III), potassium hexacyanoferrate (II) trihydrate, and nafion as well as human plasma were purchased from Sigma-Aldrich (St. Louis, MO, USA). BCA protein assay kits (Pierce™ biotin quantification kit) and streptavidin-coated microwell plates were purchased from Thermo Fisher Scientific (Rockford, IL, USA). The horseradish peroxidase (HRP)-conjugated anti-M13 antibody was purchased from sino biological (Beijing, China). The Ph.D.TM-12 phage display peptide library kit was purchased from New England Biolabs (Ipswich, MA, USA). Lithium fluoride (LiF, − 300 mesh), hydrochloric acid (HCl, ≥ 37%), gold (III) chloride trihydrate (HAuCl_4_·3H_2_O ≥ 99.9%), and trisodium citrate dihydrate (Na_3_Ctr·2H_2_O) were purchased from Sigma-Aldrich Chemicals (MO, USA). Titanium aluminum carbide (Ti_3_AlC_2_ ≥ 95%) in MAX phase was obtained from Y-Cabon Ltd. (Ukraine). Unless otherwise stated, all the chemicals were of analytical grade.

### Preparation of patient samples (plasma and serum)

Blood samples extracted from eight Crohn’s patients were provided by the Biobank of Gyeongsang National University Hospital, a member of the Korea Biobank Network and tested to assess the feasibility of the developed sensor. This study was approved by the ethics review committee of the Institution Review Board, Chung-Ang University (Approval no: 1041078-202107-BR-213-01C). For the analysis, the plasma and serum in the blood samples were separated by centrifugation (3000×*g*, 20 min) and then stored at − 80 °C until further use. The fractionated plasma and serum were used directly for the electrochemical measurements without further purification. Detailed clinical characteristics of the individuals are shown in Additional file 1: Table S1.

### Biotin labeling of cathepsin B proteins

Biotinylation of the cathepsin B proteins was performed following the manufacturer’s instructions. Cathepsin B (25 µg) was reacted with 10 mM biotin (50-fold molar excess) and then incubated at 4 °C for 24 h. To remove residual reagents, the mixtures were purified using a Zeba desalting spin column (Thermo Fisher Scientific). To estimate the biotin levels quantitatively, biotinylated cathepsin B (100 μL, 250 nM) was coated on streptavidin-coated microplates at 25 °C for 1 h, and streptavidin-conjugated HRP was used to determine the biotinylated protein content. Absorbance was measured at 405 nm using the Multiskan FC microplate photometer (Thermo Fisher Scientific, MA). Further, the Pierce™ biotin quantitation kit was used to determine the biotinylation levels in the labeled cathepsin B.

### Phage display

Biotinylated cathepsin B (99 μL, 500 nM) was pre-reacted with the Ph.D.-12 random phage library (1 μL, 1.0 × 10^13^ PFU/mL) at 100 rpm for 1 h. Subsequently, 100 µL of the complex mixture was added onto a pre-washed streptavidin-coated microplate, and allowed to react at a shaking speed of 100 rpm for 10 min to facilitate specific binding between avidin and biotin. Then, 0.1 mM biotin was added as a blocking agent and allowed to react for 5 min. After removing the unbound phages and residual biotin, the plate was washed 10 times with 0.1% PBST (0.1 M phosphate-buffered saline (PBS) with 0.1% Tween 20), and the bound phages were eluted using 100 µL of 0.2 M glycine–HCl (pH 2.2) with 1 mg/mL of bovine serum albumin (BSA) solution. Finally, the eluent was neutralized with 15 µL of Tris–HCl (pH 9.1) to prevent deactivation or destruction of the desired phages. Throughout this process, the desired phages were amplified using *Escherichia coli* ER2738 making sufficient copies for the next rounds. The amplified phages were then harvested by polyethylene glycol (PEG)/NaCl precipitation (20% (v/v) PEG-8000 with 2.5 M NaCl). After every biopanning round, the phages were tittered, and the displayed peptide sequences were analyzed using the − 96 gIII sequencing primer (5ʹ-^HO^CCC TCA TAG TTA GCG TAA CG-3ʹ).

### ELISA measurements

A 96 well streptavidin-coated microplate was washed three times with PBS, and the biotinylated cathepsin B was immobilized on it with mild shaking at 25 °C for 1 h. The unbound proteins were removed and blocked with a blocking buffer (5% BSA in NaHCO_3_) at 25 °C for 1 h. The wells were washed six times with 0.1% PBST, and 100 µL of the amplified phages (10^11^ PFU/mL), i.e., the capture receptors, were added and incubated for 1 h at 25 °C. After washing six times with PBST again, the HRP-conjugated anti-M13 monoclonal antibodies (1:5000 dilution with blocking buffer), i.e., the detecting antibodies, were added and incubated at 25 °C for 1 h. The residual solution was discarded, and the microplate was washed again with the same buffer. Freshly prepared HRP substrate (ABTS) was added, and the absorbance was measured at 405 nm using a Multiskan FC microplate photometer (Thermo Scientific, Waltham, MA, USA).

### Electrochemical measurements

A conventional three-electrode system including an Au working electrode (diameter: 5 mm, surface area: 19.6 mm^2^), a Pt counter electrode, and an Ag/AgCl reference electrode, was used for the electrochemical measurements. The EIS, CV, and SWV measurements were conducted in the presence of 2.5 mM ferro/ferricyanide in 1 M KNO_3_ using a CHI 660E instrument (CH Instruments, Austin, TX, USA) at 25 °C. The EIS measurements were carried out by applying a 10 mV signal with a frequency ranging from 0.01 Hz to 100 kHz. The CV tests were performed between − 0.6 and 0.6 V (scan range) at a scan rate of 100 mVs^−1^. The SWV tests were carried out in the potential range of − 0.4 to 0.6 V with an amplitude of 5 mV, frequency of 10 Hz, and a scan interval of 4 mV. The relative current change (*ΔI*%) was obtained by considering that the peak current decreased after the immobilization of the peptides and/or phages and cathepsin B protein interaction using the following equation:

1 $$\Delta I\% = \left( {I_0 - I} \right)/I_0 \times 100$$

where *I*_0_ refers to phage-on-a-sensor, peptide-on-a-sensor, or peptide-on-a-AuNPs/MXene sensor and/or peptide oxidation current before cathepsin B introduction, and *I* refers to Au-protein oxidation current after cathepsin B introduction.

### Statistical analysis

Two-way ANOVA was used to determine the significance of the peptide binding affinities (GraphPad Software Inc, La Jolla, CA). Additionally, the Student’s *t*-test was used to compare the *ΔI*% values.

## Results and discussion

### Identification and characterization of cathepsin B-specific high-affinity peptides

The M13 polyvalent phage display library was biopanned for the selection of dodecamer high-affinity peptides. The phage library used in this study is commercially available and contains random 12-mer peptide sequences that are fused to pIII genes. The labeled biotin level of biotinylated cathepsin B protein was confirmed using HRP-conjugated streptavidin and ABTS at 405 nm (Additional file 1: Fig. S1a). Further, the biotin quantity was analyzed using a biotin quantitation kit. The biotinylated cathepsin B was added to a mixture of 4ʹ-hydroxyazobenzene-2-carboxylic acid, biotinylated HRP, and BSA (biotinylated HRP and BSA were used as the controls) and the biotin ratio of each protein was calculated (Additional file 1: Fig. S1b and c). Four rounds of biopanning were carried out for cathepsin B, and the individual phage clones obtained from each biopanning round were randomly sequenced at the pIII region encoding the phage library peptides; the biopanning yield is shown in Additional file 1: Table S2. Selection was based on the most commonly appearing peptides in each round, whereby three phage clones were chosen for the cathepsin B binding experiments. In two biopanning rounds, only one of the chosen sequences (CTSB 2–3) contained a DG motif at the first and second positions, while two sequences (CTSB 4-1 and CTSB 4-9) in four rounds of biopanning contained PS motifs at the fifth and sixth, and sixth and seventh positions, respectively (Additional file 1: Table S3).

**Fig. 2:**
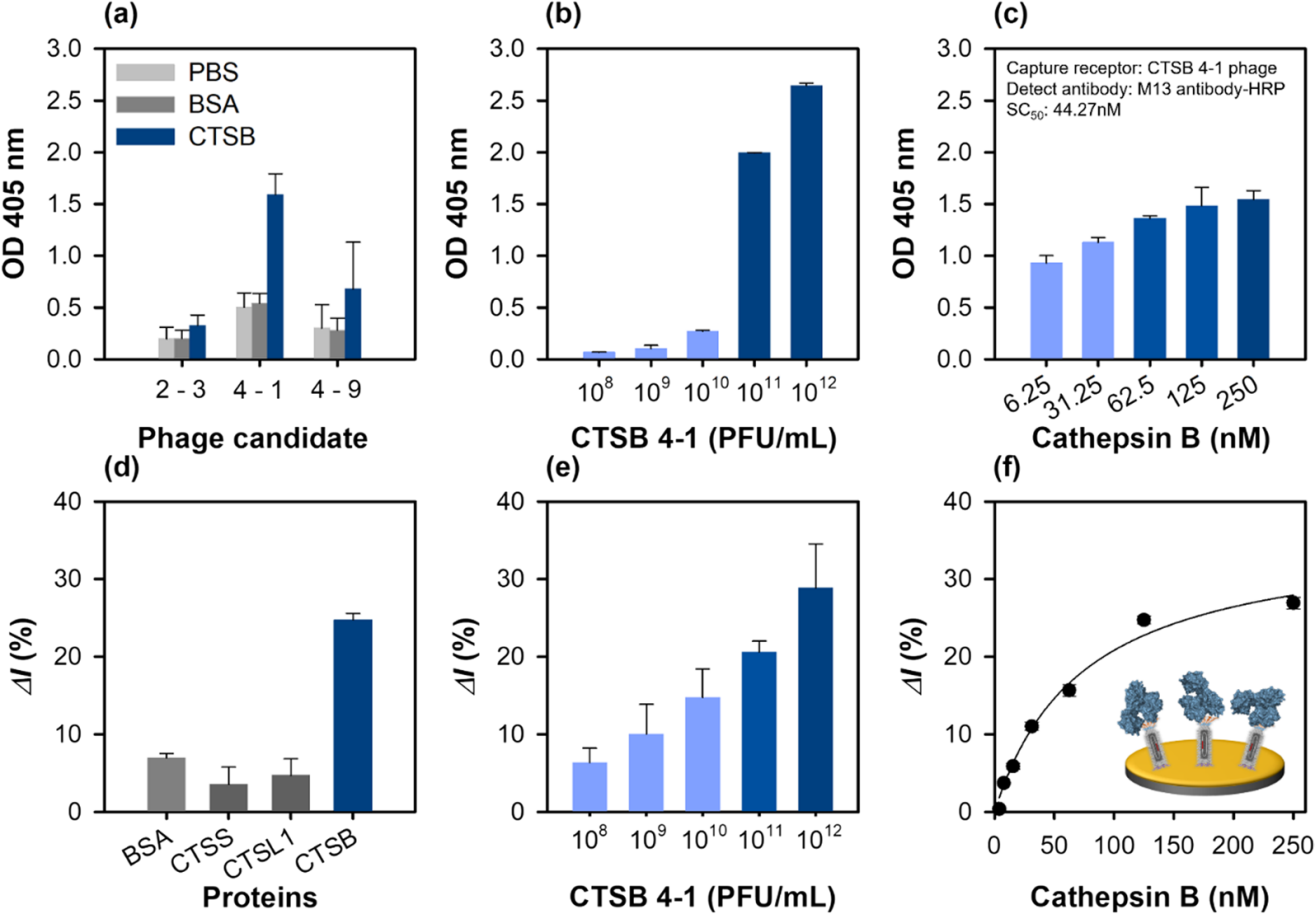
Characterization of the cathepsin B-binding phage particles using ELISA at 405 nm and SWV in 1 M KNO_3_ with 2.5 mM [Fe(CN)6]^3−/4−^. **a** Relative binding affinities of the three newly identified phage clones (~ 10^11^ PFU/mL). BSA was used as the control. **b** Effect of CTSB 4-1 concentration on the binding interactions; concentrate range: 10^8^ to 10^12^ PFU/mL. All the measurements were performed in triplicate, and the error bars represent standard deviations. **c** Relative binding affinities of the selected phage (CTSB 4-1) clones at different cathepsin B concentrations ranging from 6.25 to 250 nM. **d** Specificity of the CTSB 4-1 phage (~ 10^12^ PFU/mL)-based electrochemical sensor using various proteins (BSA, CTSS, CTSL1, CTSB, 125 nM). **e** Effect of the CTSB 4-1 phage concentration ranging from 10^8^ to 10^12^ PFU/mL on the binding interactions. **f** Determination of the binding constant of the selected CTSB 4-1 phage

The binding affinities of the selected phage clones for the immobilized cathepsin B were tested by ELISA. For these experiments, 250 nM of cathepsin B or the same amount of BSA as a negative control was immobilized on a well, followed by 10^11^ PFU/mL of phage addition. The relative binding affinity of each phage was detected using HRP-conjugated M13 antibodies. Two phage clones (CTSB 4-1 and CTSB 4-9) exhibited strong cathepsin B binding, whereas the binding affinity of CTSB 2-3 was much lower (Fig. 2a). Binding to cathepsin B could be related to the PS motif because both the phages (CTSB 4-1, and CTSB 4-9) contained a PS motif in their sequences and could bound to the same epitope on cathepsin B. Interestingly, the CTSB 4-1 phage clones exhibited a dose-dependent binding affinity with increasing cathepsin B concentration from 3.906 to 250 nM, indicating a dose–response binding to cathepsin B (Fig. 2b). The CTSB 4-1 clone was the only phage with a high binding affinity to cathepsin B, motivating further characterization of its binding interaction to confirm its specific binding to cathepsin B with increasing cathepsin B concentration (Fig. 2c).

Some researchers have reported M13 peptide-displaying phages instead of antibodies as the recognition elements or chemical scaffolds in biosensing platforms [14, 17] because of their robust, easy, and cost-effective mass production. As proof of concept, a phage sensor was developed to detect cathepsin B. The CTSB 4-1 phage particle identified by phage display was immobilized on an Au electrode pre-functionalized with the MUA-EDC/NHS coupling chemistry, and the detection performance of the fabricated phage sensor was examined by CV, EIS and SWV. Additional file 1: Fig. S2a–c shows the CV, EIS and SWV results obtained for the phage-immobilized electrodes after each step of preparation. In the CV and SWV data, the peak potential shifted after the phage immobilization step because the presence of a biomolecular component can shift the potential of a phage-coated Au electrode [33]. Additionally, the X-ray photoelectron spectroscopy (XPS) survey spectra indicated that the intensities of the peaks of Au (Au 4p_3_, Au 4d_3_, Au 4d_5_, and Au 4f) decreased after the functionalization step performed using the MUA-EDC/NHS coupling, and the peaks almost disappeared after the phage binding. These results were in stark contrast to those of the bare Au electrode (Additional file 1: Fig. S2d). Furthermore, the O 1s, N 1s, and C 1s peak intensities increased significantly upon phage functionalization of the electrode.

The electrochemical response of the phage sensor for cathepsin B detection was evaluated by the specificity analysis of the developed phage. In biosensing, specificity is a critical factor for field testing. Three different cathepsin derivatives, cathepsin S (CTSS), L1 (CTSL1), B and BSA (control) were added to the developed phage sensor, and the binding response was investigated by SWV. As shown in Fig. 2d, among the three cathepsin derivatives, the developed phage sensor exhibited favorable binding to cathepsin B. The high specificity of the peptide-displaying phage-tethered biosensor to cathepsin B indicated an accurate biopanning for isolated affinity peptides against cathepsin B. In the control experiment, the phage sensor did not specifically bind to BSA, as expected. The relative binding affinity of the phage-on-a-sensor with different phage concentrations was measured. A dynamic linear relation was observed with an increase in the phage concentration up to 10^12^ PFU/mL (Fig. 2e). The developed phage sensor could be an effective electrochemical sensing platform, as it provides an active surface area on the sensor layer. The binding constants of the developed phage sensor and the relative current change (*ΔI*%) with different cathepsin B concentrations were measured by SWV. The binding constant of the developed phage sensor was 73.73 ± 13.42 nM, and its linear dynamic response was observed in the 0–62.5 nM range (Fig. 2f).

### Chemical synthesis of affinity peptides and their analytical characterization for cathepsin B detection

The CTSB 4-1 phage was selected for sensor development during the biopanning and for the characterization of the affinity peptides using ELISA. The WDMWPSMDWKAE sequence identified in four rounds of the biopanning was used as the chemical motif for rational designing the synthetic peptide derivatives (Additional file 1: Table S4). In CTSB BP1, WDMWPSMDWKAE was attached to the biotin at the C-termini for site-specific immobilization on the streptavidin-coated Au electrodes, which could bind two or three biotin-labelled peptides with each streptavidin. CTSB BP2 was designed to analyze the effect of polar amino acid residues on the binding interactions. Three amino acids in CTSB BP1 were substituted with non-polar residues to generate CTSB BP2 with an amino acid sequence of WGMWPGMGWPAGK-biotin. Specifically, Asp and Ser in the second and sixth positions of CTSB BP1, respectively, were substituted with Gly. CTSB BP3 was synthesized to investigate the effect of polar amino acid residues on the binding interactions with an amino acid sequence of SSTTNSNSTSNTK-biotin. In CTSB BP3, all the CTSB BP1 non-polar amino acid residues were changed to polar residues via substitutions such as the replacement of Trp with Ser at the first position of CTSB BP1. CTSB BP4, with the amino acid sequence of HRHRRHRHRKHHK-biotin, contained all positively charged amino acid residues. In this case, each amino acid on CTSB BP1 was substituted to obtain CTSB BP4. It was used to investigate the effect of positively charged amino acid residues on the binding. To synthesize CTSB BP5 with an amino acid sequence of DDEDDEEDDEDEK-biotin, the amino acids on CTSB BP1 were substituted with negatively charged residues. CTSB BP5 was synthesized to analyze the effect of negatively charged amino acid residues on binding interactions.

**Fig. 3:**
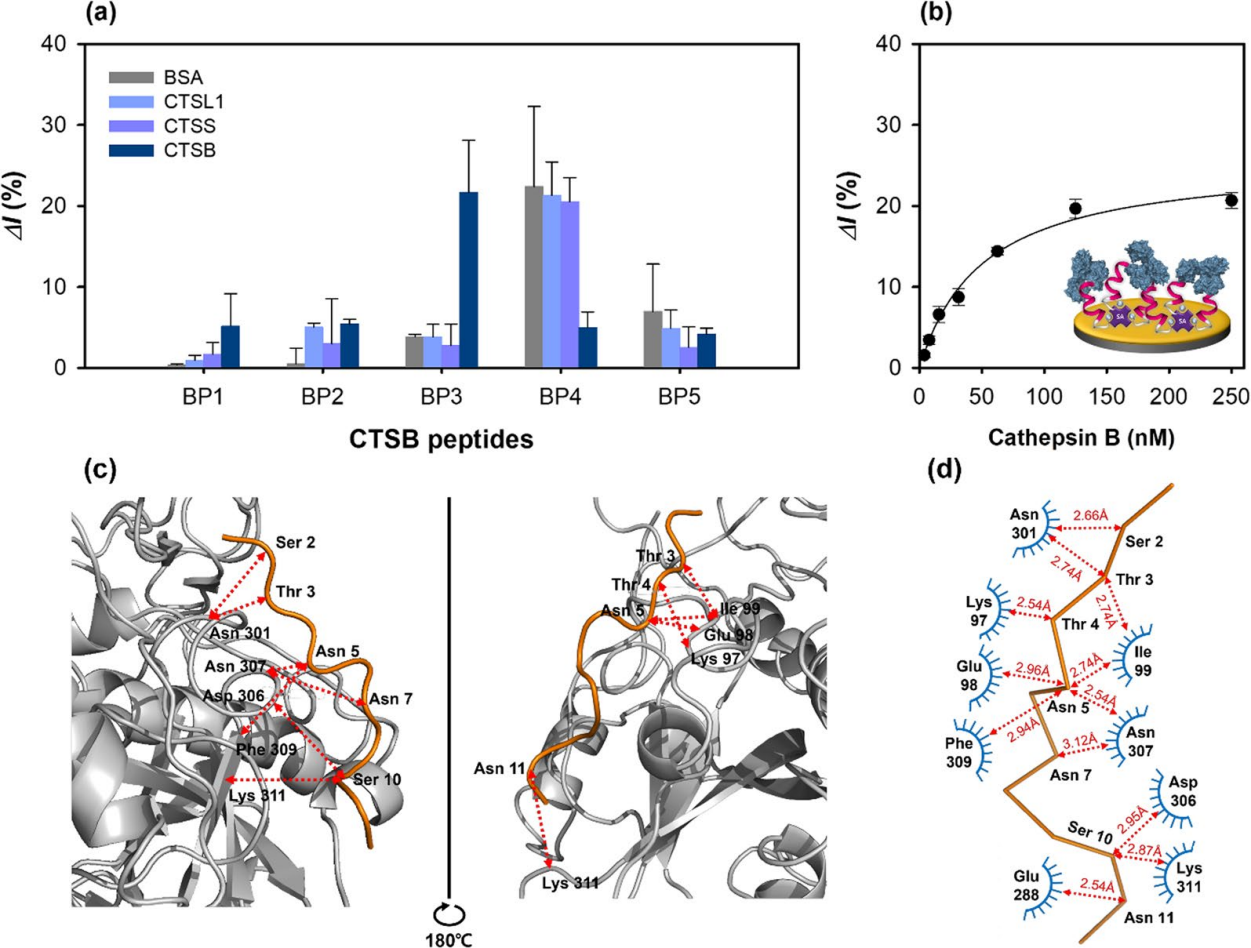
Characterization of peptide-based electrochemical sensors using SWV at 1 M KNO_3_ with 2.5 mM [Fe(CN)_6_]^3−/4−^. **a** Specificity of peptide-on-a-sensor using various proteins (BSA, CTSS, CTSL1, CTSB, 125 nM). **b** Determination of the binding constant of the peptide-on-a-sensor, along with the results of molecular docking between CTSB BP3 and cathepsin B. **c** Front and side views of the molecular docking results (docking of the CTSB BP3 peptide with the cathepsin B protein). **d** Interaction network between the CTSB BP3 peptide and the cathepsin B protein. The arrow indicates the contact distance (Å)

To select potential high-affinity peptides from the phage clones, the five synthetic peptides were immobilized on the Au electrode via streptavidin–biotin and MUA-EDC/NHS coupling methods. Each preparation step of the peptide sensor was recorded using CV, EIS, and SWV (Additional file 1: Fig. S3a–c), and the relative binding affinities for cathepsin B were measured by SWV. Further, the intensities of the peaks of O 1s, N 1s, and C 1s increased after the functionalization steps, compared with those observed for bare Au; moreover, the Au peak intensities decreased (Additional file 1: Fig. S3d). The different synthetic peptides (CTSB BP1–BP5, 50 μM) were washed with pure distilled water and immobilized on the streptavidin-modified Au surface layer and incubated with the cathepsin B protein (125 nM) for 1 h. In these experiments, BSA was used as a control, and the three cathepsin derivatives, viz. cathepsin B, CTSS, and CTSL1 were used to determine the binding affinities of the peptides to different proteins. CTSB BP3 exhibited the highest binding affinity to cathepsin B, the primary target protein in the biopanning (Fig. 3a). This result was in agreement with the hypothesis, indicating an accurate identification of the high-affinity peptides under the well-controlled experimental conditions. Unlike CTSB BP3, CTSB BP4 had non-specific binding affinity for BSA, CTSL1, and CTSS with a much lower binding affinity for cathepsin B than for CTSB BP3. This low binding affinity can be attributed to two reasons. First, the positively charged amino acids in the HRHRRHRHRKHHK-biotin amino acid sequence of CTSB BP4 could mediate the peptide–cathepsin B binding interactions. Second, the five Arg residues in CTSB BP4 could be protonated with a higher charge than that of the aliphatic amino acids, leading to weak binding. The relative binding affinity of CTSB BP1 for all the tested proteins was comparable to those of CTSB BP2 and CTSB BP5; however, it did not bind to cathepsin B. Thus, CTSB BP3 was selected for the feasibility tests and characterization studies.

The feasibility of the selected peptide (CTSB BP3) tethered on a sensor for cathepsin detection was analyzed by SWV measurements at different peptide concentrations (5–100 μM) (Additional file 1: Fig. S4a). The relative current change (*ΔI*%) gradually increased up to 50 μM and then slightly decreased at 100 μM peptide concentration (Additional file 1: Fig. S4b). Therefore, 50 μM peptide concentration was selected as the optimal concentration for the characterizations. The change in relative current with increasing cathepsin B concentration, analyzed using SWV (Fig. 3b), exhibited a sigmoidal curve. From these results, the binding constant, *K*_d_ was found to be 50.78 ± 7.57 nM.

To identify and elucidate the possible binding sites of the CTSB BP3 peptide with cathepsin B, molecular docking was conducted using the CABS-dock software, and the corresponding docking results are presented in Additional file 1: Table S5. The front and side views of the docking results showed that the CTSB BP3 peptide bound linearly to the cathepsin B protein (Fig. 3c). The binding interfaces of the amino acid residues of the peptide (Ser 2, Thr 3, Asn 5, Ser 10, and Asn 11) interacted with the residue random coil (Glu 98, Ile 99, Asn 301, Asp 306, Asn 307), helix (Lys 97), and β-strand (Glu 288, Phe 309 and Lys 311) under a contact cutoff of 3 Å (Fig. 3d). The interaction between the polar amino acid residues (Ser, Thr, Asn) of the peptide and the electrically charged (Lys, Glu, Asp) and hydrophobic (Ile, Phe) amino acid could be one of the reasons for the high binding affinity. It should be noted that molecular docking is just a simulation without any experimental verification.

### Fabrication and characterization of the peptide-on-a-AuNPs/MXene sensor

#### Synthesis and characterization of AuNP–MXene nanocomposites

A phage-on-a-sensor and a peptide-on-a-sensor were developed and their cathepsin B detection performances were evaluated. However, our aim was to develop a more advanced electrochemical sensor with high sensitivity. Efficient and advanced electrochemical sensors can be fabricated by incorporating nanomaterials; they exhibit good electrical conductivity and chemical stability and thus can be operated as biosensors. AuNPs (20 nm in size) in combination with MXene (single-layered form) were used to fabricate the peptide-on-a-AuNPs/MXene sensor, whose cathepsin B detection performance was subsequently evaluated. We expected that the introduction of AuNPs–MXene would significantly improve the electron transfer between the electrode and the electrolyte solutions, compared with other electrode materials because of the large surface area and porosity of the AuNPs. The AuNPs-embedded MXene was synthesized by selective etching of Al from the MAX phases using chemicals depending on the different AuNPs:MXene concentration ratios (0.5–5 mL Au, 50 mg/mL single-layered MXene). The morphology and composition of the synthesized AuNPs–MXene composites were analyzed by scanning electron microscopy and transmission electron microscopy. The AuNPs were attached to the Ti_3_C_2_F MXene layers in the AuNPs–MXene composites (Additional file 1: Fig. S5). The AuNPs were synthesized uniformly in spherical shapes through the chemical reduction of AuCl_4_^−^ with a mean particle diameter of 13.21 ± 1.55 nm (Additional file 1: Fig. S6a and b). Further, a single layer of the 2D Ti_3_C_2_F MXene was fabricated in a single step using HCl and LiF salts (Additional file 1: Fig. S6c and d). The atomic ratios of the composites were determined by energy-dispersive X-ray spectroscopic measurements (Additional file 1: Fig. S7), and the Ti:C:F ratio was found to be 3.01:1.88:1.00, which well-matched the stoichiometric ratio of the Ti_3_C_2_F MXene (Additional file 1: Fig. S7a). Additionally, the corresponding elemental mapping images confirmed the compositional distribution of the AuNPs–MXene composites (Additional file 1: Fig. S7b–e).

Next, the crystallographic structures and phase purity of the AuNPs–MXene composites were evaluated by X-ray diffraction (XRD). As shown in Additional file 1: Fig. S8a, the peaks at 2θ values of 9.02°, 18.19°, 30.63°, and 60.65° in the XRD patterns could be ascribed to the (002), (006), (008), and (110) planes, respectively, indicating the crystalline structure of the MXene [33]. Additionally, four diffraction peaks at 38.18°, 44.52°, 64.64°, and 77.58°, corresponding to the (111), (200), (220), and (311) planes were detected in the AuNP–MXene composites, indicating the face-centered cubic lattice of the AuNPs [34]. These diffraction peaks indicated that the AuNP attachment had no effect on the MXene crystal structure. The formation of AuNPs–MXene composites was further confirmed by Fourier transform infrared spectroscopy, as shown in Additional file 1: Fig. S8b. The adsorption peaks at 3743.91, 1646.61, 1037.29, and 675.22 cm^−1^ were associated with the O–H, C=C, C–F, and Ti–O stretching vibrations of the MXene layer [35].

#### Verification of the peptide-on-a-AuNPs/MXene sensor performance

**Fig. 4:**
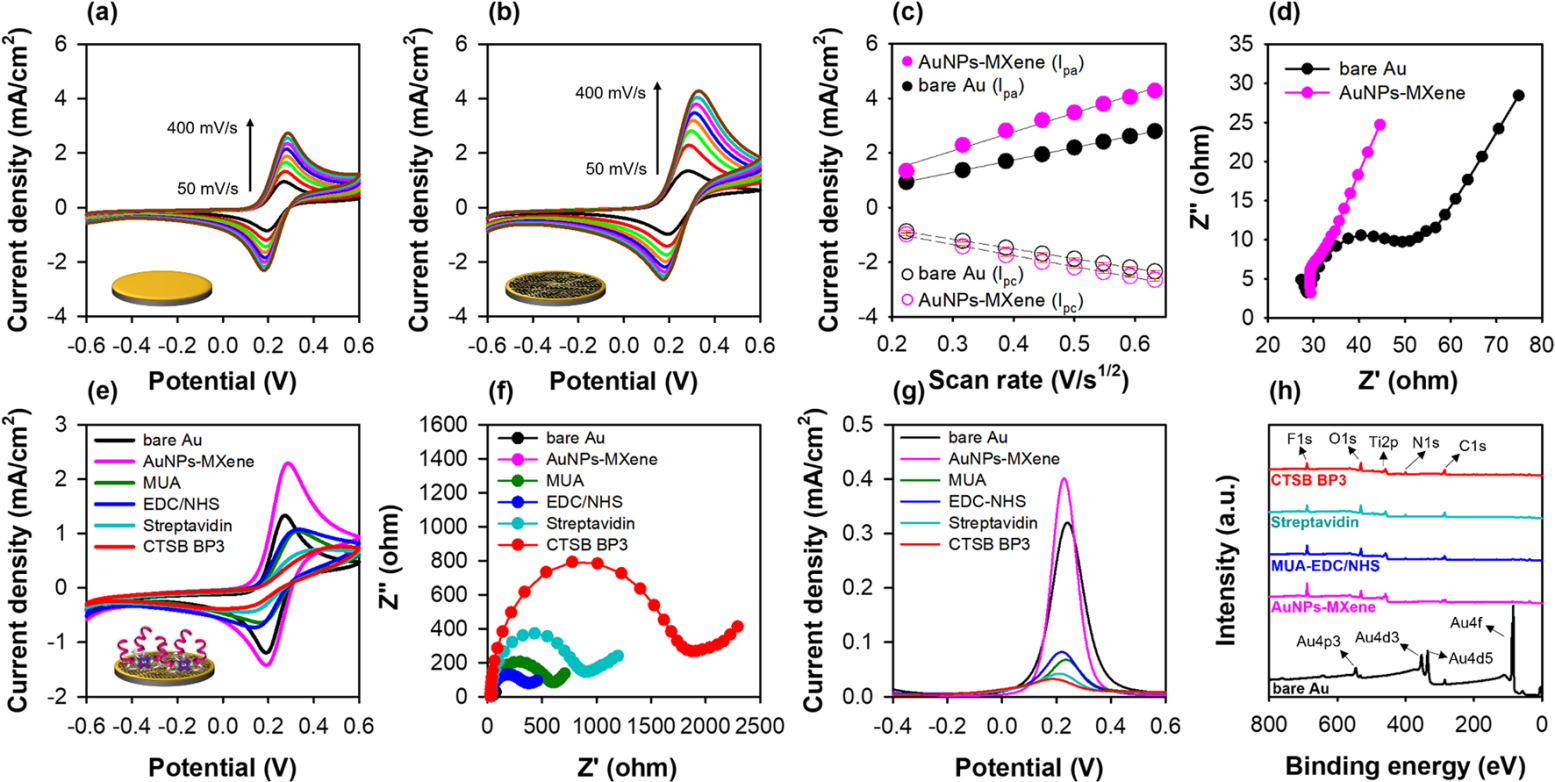
Characterization of AuNPs–MXene fabricated electrode. **a**–**c** Effect of scan rate (50 to 400 mVs^−1^) for bare Au and AuNPs–MXene fabricated Au electrodes determined by CV at 1 M KNO_3_ with 2.5 mM [Fe(CN)_6_]^3−/4−^ and Randles-Sevcik plot. In **c**, all the measurements were done in triplicate, and the error bars represent standard deviations. **d** Electrical transfer resistance of each electrode (Au, AuNPs–MXene) measured by EIS. **e**–**g** CV, EIS, and SWV responses for each preparation step of the peptide-on-a-MXene sensor. **h** XPS spectra of bare Au, Au@AuNPs–MXene, Au@MUA-EDC/NHS, Au@streptavidin and Au@CTSB BP3 peptide

After the AuNPs-embedded MXene nanocomposite fabrication, the peptide-on-a-AuNPs/MXene sensor was formed by immobilizing the peptide on the nanocomposite layer. Prior to peptide immobilization, the AuNPs-embedded MXene was attached to the Au electrode sensor layer using 0.1% nafion. As shown in Additional file 1: Fig. S9a and b, 4 mg/mL of the AuNPs-embedded MXene on the Au electrode exhibited a higher conductivity compared with the other concentrations. The active surface areas of bare Au and Au@AuNPs–MXene were extracted from the relationship between the anodic peak current and scan rate of their CV responses using the Randles–Sevick equation (Fig. 4a–c). The active surface area was evaluated to be 0.071 cm^2^, which is higher than that of the bare Au (0.041 cm^2^), AuNPs (0.045 cm^2^) and MXene (0.049 cm^2^) electrodes at 100 mV/s of scan rate (Additional file 1: Table S6). Furthermore, the electrode charge transfer resistance (R_ct_) reduced because of the increasing permeability of the redox probes (Fig. 4d). After optimization, CTSB BP3 was immobilized on the functionalized MXene sensor, and its preparation steps were analyzed by electrochemical measurements (Fig. 4e–g) and XPS (Fig. 4h). The results of CV, EIS and SWV for the peptide-on-AuNPs/MXene sensor showed similar responses for each step, and the changes in current and resistance for peptide immobilization were higher than those for the other sensors (phage and only peptide) because of the increased surface area which could bind with the peptide compared with that of the bare Au electrode. Further, the peaks of Au almost disappeared after the fabrication of AuNPs–MXene, whereas the F 1 s and Ti 2p peaks appeared for the Au@AuNPs–MXene electrode. Moreover, the peak intensities of O 1 s, N 1 s, and C 1 s gradually increased according to the peptide immobilization steps.

**Fig. 5:**
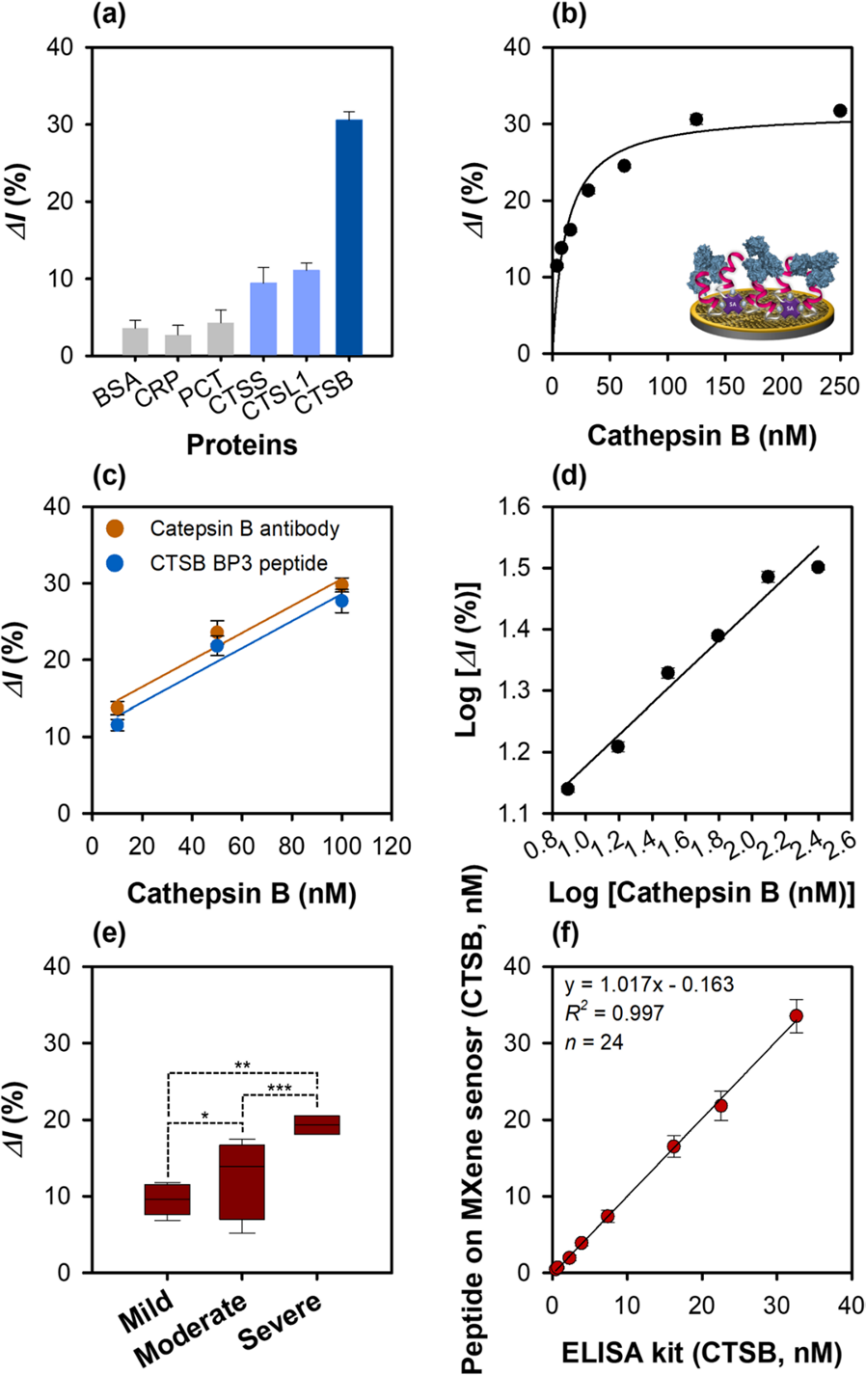
Characterization of peptide-on-a-AuNPs/MXene sensor. **a** Specificity of peptide-on-a-AuNPs/MXene sensor using various proteins (BSA, CRP, CPT, CTSS, CTSL1, CTSB, 125 nM). **b** Determination of the binding constant of peptide-on-a-AuNPs/MXene sensor. **c** Comparison of binding affinity between cathepsin B polyclonal antibody (100 μg/mL) and CTSB BP3 peptide (100 μg/mL: ~ 50 μM) on Mxene sensor. **d** Linear graph of peptide-on-a-AuNPs/MXene sensor. **e** Comparison of CTSB concentration in Crohn’s disease patient samples (*: *p* > 0.05, **, ***: *p* < 0.05). **f** Linear correlation between commercially available ELISA and peptide-on-AuNPs/MXene sensor for CTSB concentration determination in eight human plasma, and serum samples. The SWV measurements were performed in 1 M KNO_3_ containing 2.5 mM [Fe(CN)_6_]^3−/4−^. All the measurements were done in triplicate, and the error bars represent standard deviations. In the case of Fig. 5e, each sample was analyzed three times, resulting in a total of 24 measurements

The change in the current for different proteins (BSA, C-reactive protein (CRP), procalcitonin (PCT), CTSS, and CTSL1) which are related to inflammatory response was monitored by SWV to test the cathepsin B detection feasibility of the peptide-on-a-AuNPs/MXene sensor. The sensor exhibited the highest binding affinity for the target protein cathepsin B, and very low binding affinities for CTSS and CTSL1, indicating its specificity and selectivity for cathepsin B. As expected, no specific binding affinity was observed for BSA, CRP, and PCT (Fig. 5a). Additionally, the binding behavior of the sensor dependent on peptide concentration in the 5–100 μM range was analyzed by SWV (Additional file 1: Fig. S10a). The relative current change (*ΔI*%) gradually increased up to the peptide concentration of 50 μM and then slightly decreased at 100 μM (Additional file 1: Fig. S10), showing a sigmoidal curve as shown in Fig. 5b. This may probably due to the inter/intra-interaction of individual immobilized peptide or steric hindrance on the electrode, resulting in the decrease of binding affinity for cathespsin B. Therefore, 50 μM peptide concentration was selected as the optimal concentration for the subsequent analyses. The binding constant of the peptide-on-a-AuNPs/MXene sensor was found to be 11.64 ± 2.88 nM.

### Determination of electrochemical response of the sensors for cathepsin B detection

To compare the cathepsin B detection performances, three different sensors were developed, and the relative current change (*ΔI*%) shown by these sensors with increasing cathepsin B concentration from 3.9 to 125 nM was observed using SWV. The binding affinity of all the developed sensors increased proportionally with increasing protein concentration. The binding constants (*K*_d_) of the three developed sensors, viz. phage-on-a-sensor, peptide-on-a-sensor, and peptide-on-a-AuNPs/MXene sensors were calculated to be 73.73 ± 13.42, 50.78 ± 7.57, and 11.64 ± 2.88 nM, respectively. Furthermore, the cathepsin B detection ability of the peptide-on-a AuNPs/MXene sensor was similar to that of the cathepsin B antibody-immobilized MXene sensor (Fig. 5c).

Their LOD and limit of quantitation (LOQ) were calculated using the following equations:

2 $${\text{LOD}} = 3 \times \rm{\delta }/{\text{s}}$$

3 $${\text{LOQ}} = 10 \times \rm{\delta }/{\text{s}}$$

where δ and s are the standard deviation of the y-intercept and slope of the standard curve, respectively. As shown in the Fig. 5d, Additional file 1: Fig. S11 and Table 1, the LOD and LOQ of the peptide-on-a-AuNPs/MXene sensor exhibited the most sensitive cathepsin B detection were 0.18 and 0.59 nM, respectively, which were determined using the equation: y = 0.261x + 0.912, where R^2^ = 0.99, δ = 0.015, and s = 0.261. The LOD and LOQ of the phage-on-a-sensor for cathepsin B detection were 0.62 and 2.07 nM, respectively, obtained using the equation: y = 0.093x − 0.5392, where R^2^ = 0.96, δ = 0.192, and s = 0.093. The corresponding values for the peptide-on-a-sensor were 0.33 and 1.11 nM, respectively, which were derived using the equation: y = 0.626x − 0.046, where R^2^ = 0.97, δ = 0.069, and s = 0.626. Among the three developed sensors, the peptide-on-a-AuNPs/MXene sensor exhibited the most sensitive cathepsin B detection.

**Table 1:** Comparison of sensor performances for cathepsin B detection

Sensor performance	Phage-on-a-sensor	Peptide-on-a-sensor	Peptide-on-a-AuNPs/MXene sensor
LOD (nM)	0.62	0.33	0.18
LOQ (nM)	2.07	1.11	0.59
*K*_d_ (nM)	73.73 ± 13.42	50.78 ± 7.57	11.64 ± 2.88
Average recovery (%)	91.05–93.58	96.03–99.43	94.52–101.16

### Reproducibility and stability of the cathepsin B sensors

Reproducibility and recovery of biosensors are considered key parameters for commercialization. Therefore, the reproducibility and recovery of the developed sensors were investigated with different cathepsin B concentrations (31.3‒125 nM) spiked in human plasma samples; these analyses were conducted using SWV. The acceptable coefficient of variation and percentage recoveries for the developed sensors were remarkable (Additional file 1: Table S7). The recovery percentage and coefficient of variation (%) were in the range of 96.03–99.43%, and 1.30–6.99, respectively, for the peptide-on-a-sensor, whereas they were in the range of 91.05–93.58%, and 3.85–8.89 for the phage-on-a-sensor. Thus, the recovery percentage and CV (%) values of the peptide-on-a-sensor were much higher than those of the phage-on-a-sensor, indicating that the phage-on-a-sensor could be used as a building block, i.e., as an alternative recognition element. However, its application in clinical testing is not recommended. In contrast, the recovery percent and coefficient of variation (%) values of the peptide-on-a-AuNPs/MXene sensor were in the range of 94.52–101.16%, and 1.68–6.04, respectively, indicating a reproducible and accurate cathepsin B detection ability of this sensor in a complex biological sample (human plasma).

Another vital parameter of a biosensor is its stability. Therefore, the stability of the three different sensors was monitored for 6 days after peptide immobilization on each sensor layer. For the phage-on-a-sensor, the relative current change (*ΔI*%) decreased by 3.3% on day 1, and gradually dropped by 34.7% from its initial value after the sensor was stored overnight at 4 ℃ (Additional file 1: Fig. S12a). The peptide-on-a-sensor signal was stable on day 1, but its relative current change (*ΔI*%) decreased by 17.5% on day 2 and further gradually decreased to 43.8% on day 6 (Additional file 1: Fig. S12b). Interestingly, under similar conditions, the relative current change (*ΔI*%) of the peptide-on-a-AuNPs/MXene sensor remained stable up to day 4, and finally dropped to 29.4% from its initial value (Additional file 1: Fig. S13c), indicating high sensor stability. This could be due to the introduction of peptides on the single-layered MXene surface resulting in an increased electrostatic interaction (attraction or repulsion) which firmly held the peptides between each MXene layer, improving the structural stability and affinity of the peptides. The analytical cathepsin B detection performances of the developed sensors are compared in Additional file 1: Table S8 along with other detection methods.

### Electrochemical sensing with real patient samples

Real testing with liquid biopsy samples such as blood, nucleic acid, and protein derived from human patients is essential for biosensor performance evaluation. Therefore, as proof-of-concept, SWV measurements of diluted crude plasma (1:100 in PBS buffer, *n* = 5) and serum (1:100 in PBS buffer, *n* = 3) obtained from patients suffering from Crohns’s disease (*n* = 8 in total) were performed. The current change observed in the SWV data is shown in Fig. 5e. Interestingly, statistically significant differences in the relative current change (*ΔI*%) were observed among the mild, moderate, and severe groups using the developed electrochemical sensor (as analyzed with two-way ANOVA). The change in current for both the moderate and severe groups of patients was significantly different (*p* < 0.05) from that of the moderate group (*p* > 0.05), indicating that the expression level of cathepsin B could be highly patient-dependent. Cathepsin B was up-expressed in both the moderate (up to 16 nM) and severe (up to 32 nM) groups, while the cathepsin B concentration was down to 0.7 nM in the mild group. The following order of cathepsin B levels was obtained: severe group > moderate group > mild group. This result is consistent with those of previous reports [11, 36] on cathepsin B expression in different stages of Crohn’s disease and ulcerative colitis disease. Further, this result also suggests a good correlation between increased cathepsin B concentration and a variety of diseases [37]. As shown in Fig. 5f and Additional file 1: Table S9, the cathepsin B levels measured by the fabricated peptide-on-a-AuNPs/MXene sensor were correlated with those estimated by the ELISA with good recoveries (86–102%) and %RSDs (< 11%) in all the tested cases. Although a small number of patient samples were tested, the applicability of the developed peptide sensor was comparable to the reference (commercially available ELISA). Thus, the developed electrochemical peptide sensor exhibited good detection performance with clinical patient samples and could distinguish different stages of Crohn’s disease.

## Conclusion

In this study, a novel biosensor was developed and characterized for the electrochemical detection of cathepsin B based on affinity peptides as alternative recognition elements. The M13 phage library was biopanned to identify high-affinity peptides specific for cathepsin B. The binding affinities and efficacies of the developed sensor were characterized by ELISA and through electrochemical experiments (CV, EIS and SWV). In the phage-on-a-sensor, as proof-of-concept, the phage particles were attached to an Au electrode via the MUA-EDC/NHS chemistry. The particles exhibited high biocompatibility and stability, thereby fulfilling the requirements for potent bioanalytical applications. Through a rational design and chemical synthesis of peptides away from the phage particles selected peptides were used for the construction of the peptide-on-a-sensor system. The developed peptide-on-a-sensor system exhibited good electrochemical sensing performance. Subsequently, AuNPs were introduced on the MXene layer to develop a more advanced electrochemical sensor for cathepsin B detection. The fabricated peptide-on-a-AuNPs/MXene sensor exhibited a low LOD (0.18 nM), low LOQ (0.59 nM), high recovery percentage (94.5–101.2%), and high stability (four days) as well as enabled a highly accurate detection of cathepsin B, even in complex biological samples. The clinical applicability of the peptide-on-a-AuNPs/MXene sensor was evaluated via cathepsin B detection in Crohn’s patient samples. The sensor exhibited an accurate and sensitive cathepsin B detection with a good recovery (86–102%) and %RSD (< 11%) and could distinguish between the different stages of Crohn’s disease. Furthermore, cathepsin B concentrations measured by the developed sensor correlated well with those estimated by the commercially available ELISA kit. Although our developed sensor demonstrated a high specificity and good recovery for cathepsin B, further investigations are required before it can be applied to create a peptide sensor for real clinical testing. First, the use of more functional peptides obtained via virtual re-screening with additional modifications and introduction of other nanomaterials is a challenging task. Second, the rational design of a protease-resistant peptide probe is required to minimize the inaccessibility of liquid biopsy or complex solutions in the presence of various proteases. One limitation of our electrochemical techniques including CV, EIS and SWV is the need to operate the electrode in a ferri/ferro cyanide solution (redox mediator) after the target sample is introduced on the peptide-immobilized sensor layer. This may sometimes affect the viscosity change in the electrical measurements. In summary, to the best of our knowledge, this is the first example of a rational design of affinity peptides and the development of a label-free sensing platform for cathepsin B using peptides and MXene nanocomposites. This new sensing system could be useful for the development of peptide-based sensors to detect any desired target by changing the amino acid sequence of the peptides and could be combined with various functional nanomaterials.

## Supplementary Information

**Additional file 1: Table S1.** Clinical characteristics of individuals in the detection of cathepsin B**. Table S2.** The yield of biopanning for cathepsin B. **Table S3.** Screening results of biopanning for identifying cathepsin B specific peptides via phage display. **Table S4.** The synthetic peptides for detection of cathepsin B. **Table S5.** Analysis of binding site of CTSB BP3 peptide for cathepsin B using molecular docking. **Table S6.** The electroactive surface area of bare gold and AuNPs–MXene fabricated electrodes. **Table S7.** Detection capability of developed sensors at different concentrations of cathepsin B spiked in human plasma. **Table S8.** Comparison of the analytical performance for the detection of cathepsin B. **Table S9.** Analytical results between our developed sensor and the reference ELISA with Crohn’s patient samples. **Figure S1.** The validation assay for biotinylated cathepsin B protein. (a) The biotin-labeled BSA (250 nM = 16.5 μg/mL) and CTSB (250 nM = 6.875 μg/mL) were immobilized on streptavidin coated plate, and it was confirmed using HRP conjugated streptavidin and ABTS at 405 nm. (b) To quantitate labelled biotin, the biotinylated cathepsin B was added to the mixture of HABA and avidin and biotinylated HRP and BSA were used as control. (c) Biotin ratio was calculated from biotin quantitation results, which biotin ratio for cathepsin B was about 3.745. **Figure S2.** The responses of electrochemical measurements of phage-on-a sensor. (a–c) CV, EIS and SWV responses for preparation steps of phage sensor (bare Au, Au@1 mM MUA, Au@ 400 mM/100 mM EDC/NHS, Au@4-1 phage). It was conducted at 1 M KNO_3_ with 2.5 mM [Fe(CN)_6_]^3−/4−^. (d) X-ray photoelectron spectroscopy (XPS) spectra of bare Au, Au@MUA-EDC/NHS, Au@4-1 phage. **Figure S3.** The responses of electrochemical measurements of peptide-on-a sensor. (a–c) CV, EIS, and SWV responses for preparation steps of phage sensor (bare Au, Au@1 mM MUA, Au@ 400 mM/100 mM EDC/NHS, Au@streptavidin, Au@CTSB BP3 peptide). It was conducted at 1 M KNO_3_ with 2.5 mM [Fe(CN)_6_]^3−/4−^. (d) X-ray photoelectron spectroscopy (XPS) spectra of bare Au, Au@MUA-EDC/NHS, Au@streptavidin, Au@CTSB BP3 peptide. **Figure S4.** Effect of peptide concentration for peptide-on-a sensor. (a) The current responses of different CTSB BP3 peptide concentrations (5–100 μM). (b) The current changes of peptide-on-a sensor in different CTSB BP3 peptide concentrations for cathepsin B. **Figure S5.** Morphological characterization of AuNPs-MXene composite; SEM images under (a) low and (b) high resolution; TEM images under (c) low and (d) high resolution. **Figure S6.** (a) TEM image and (b) size distribution graph of pre-synthesized AuNPs. (c) TEM image and (d) SEM image of Ti_3_C_2_F MXene. **Figure S7.** (a) EDS spectrum of AuNPs-MXene composites. (b-e) Elemental mapping images obtained from EDS for elements Ti, C, F and Au, respectively. **Figure S8.** (a) X-ray diffraction spectra and (b) FT-IR spectra of AuNPs-MXene composites (red line) and MXene (black line). **Figure S9.** Optimization of AuNP-MXene concentration and comparison of the effect of scan rate on gold electrode. (a) Chronoamperometric response of the sensor with different concentration of AuNP-MXene. (b) Square wave voltammetry analysis of the sensor with different concentration of AuNP-Mxene. **Figure S10.** Effect of peptide concentration for peptide-on-a-AuNPs/MXene sensor. (a) The current responses of different CTSB BP3 peptide concentrations (5–100 μM). (b) The current changes of peptide-on-a-AuNPs/MXene sensor in different CTSB BP3 peptide concentrations for cathepsin B. **Figure S11.** Comparison of standard curves of the developed sensors: a) Phage-on-a-sensor, b) Peptide-on-a-sensor. The change of current was measured by SWV with 1 M KNO_3_ containing 2.5 mM [Fe(CN)_6_]^3−/4−^. In phage-on-a-sensor, the titer of phages was 1 × 10^12^ PFU/mL, while the concentration of peptide used in peptide-on-a-sensor was 50 μM. All measurements were done in triplicate, and error bars represent standard deviations. **Figure S12.** Comparison of stability of the developed sensor. (a) Phage on a sensor, (b) peptide on a sensor, (c) peptide on a AuNPs/MXene sensor.

## Data Availability

Not applicable.
