# Supplementary material for: Harnessing protein sensing ability of electrochemical biosensors via a controlled peptide receptor–electrode interface
Source: J Nanobiotechnology. 2023 Mar 21;21:100. doi: 10.1186/s12951-023-01843-0 (PMC10029155; doi:10.1186/s12951-023-01843-0)
Supplement: Supplementary file 1 — Additional file 1: Table S1. Clinical characteristics of individuals in the detection of cathepsin B. Table S2. The yield of biopanning for cathepsin B. Table S3. Screening results of biopanning for identifying cathepsin B specific peptides via phage display. Table S4. The synthetic peptides for detection of cathepsin B. Table S5. Analysis of binding site of CTSB BP3 peptide for cathepsin B using molecular docking. Table S6. The electroactive surface area of bare gold and AuNPs–MXene fabricated electrodes. Table S7. Detection capability of developed sensors at different concentrations of cathepsin B spiked in human plasma. Table S8. Comparison of the analytical performance for the detection of cathepsin B. Table S9. Analytical results between our developed sensor and the reference ELISA with Crohn’s patient samples. Figure S1. The validation assay for biotinylated cathepsin B protein. (a) The biotin-labeled BSA (250 nM = 16.5 μg/mL) and CTSB (250 nM = 6.875 μg/mL) were immobilized on streptavidin coated plate, and it was confirmed using HRP conjugated streptavidin and ABTS at 405 nm. (b) To quantitate labelled biotin, the biotinylated cathepsin B was added to the mixture of HABA and avidin and biotinylated HRP and BSA were used as control. (c) Biotin ratio was calculated from biotin quantitation results, which biotin ratio for cathepsin B was about 3.745. Figure S2. The responses of electrochemical measurements of phage-on-a sensor. (a–c) CV, EIS and SWV responses for preparation steps of phage sensor (bare Au, Au@1 mM MUA, Au@ 400 mM/100 mM EDC/NHS, Au@4-1 phage). It was conducted at 1 M KNO3 with 2.5 mM [Fe(CN)6]3−/4−. (d) X-ray photoelectron spectroscopy (XPS) spectra of bare Au, Au@MUA-EDC/NHS, Au@4-1 phage. Figure S3. The responses of electrochemical measurements of peptide-on-a sensor. (a–c) CV, EIS, and SWV responses for preparation steps of phage sensor (bare Au, Au@1 mM MUA, Au@ 400 mM/100 mM EDC/NHS, Au@streptavidin, Au@CTSB BP3 peptide). It was conducted [file 12951_2023_1843_MOESM1_ESM.pdf]

Supplementary Information:

Harnessing analytical performance in electrochemically protein sensing via controlled peptide receptor-surface interface

Ji Hong Kim[a], Jae Hwan Shin[a], Bumjun Park[b], Chae Hwan Cho[a], Yun Suk Huh[b], Chang-Hyung Choi[c], Jong Pil Park[a*]

[a] Basic Research Laboratory, Department of Food Science and Technology, Chung-Ang University, Anseong 17546, Republic of Korea

[b] NanoBio High-Tech Materials Research Center, Department of Biological Engineering, Inha University, 100 Inha-ro, Incheon 22212, Republic of Korea

[c] Division of Cosmetic Science and Technology, Daegu Haany University, 1 Haanydaero, Gyeongsan, Gyeongbuk 38610, Republic of Korea

* Corresponding author:

Jong Pil Park

Tel.: +82 31 670 4703, Fax: +82 31 675 3108, E-mail: jppark@cau.ac.kr

Supplemental Note

Preparation of phage on a sensor

Preparation of a phage on a sensor was performed as follows. First, the working electrode was immersed in a piranha solution (H_2O_2:H_2SO_4=7:3, v/v) at 25 °C for 8 min for polishing and then washed with distilled water [1]. The pre-treated working electrode was dried by blowing N_2 gas. After polishing, the gold electrode was reacted with 1 mM of MUA at 25 °C for overnight with mild shaking and washed five times with pure deionized distilled water. Then, the MUA-immobilized gold electrode was immersed in 400 mM/100 mM EDC/NHS at 25 °C for 1 h [2]. After washing with distilled water, the functionalized gold electrode was incubated with peptide-displayed phages at 25 °C for 1 h. Finally, developed phage on a sensor was reacted with cathepsin B and the binding affinity was measured by SWV.

Synthesis of peptides and fabrication of peptide on a sensor

A total of five peptide derivatives were rationally designed and synthesized by solid-phase synthesis protocol (>90 % purity, Peptron, Daejeon, Korea). The synthetic peptide derivatives were dissolved using DMSO and 0.1 M PBS, and stored at -20 °C before use.

For fabrication peptide on a sensor, a gold electrode was polished similarly as in the preparation of a phage on a sensor. Then, the gold electrode was immersed in 1 mM MUA at 25 °C overnight and washed with pure deionized distilled water. The MUA-immobilized gold electrode was incubated with 400 mM/100mM EDC/NHS at 25 °C for 1 h and washed again with same distilled water. And then, 100 μg/mL of streptavidin was added to the MUA–

EDC/NHS-functionalized gold electrode at 25 °C for 2 h and washed with distilled water to remove unbound or residual reagents and biotin-labeled synthetic peptides were added and incubated at 25 °C for 1 h. Then, the peptide-functionalized sensor was incubated in 1 % BSA for 15 min to decrease non-specific binding. The developed peptide on a sensor was reacted with cathepsin B and binding affinity was observed using SWV.

Generation of gold nanoparticle-embedded MXene (AuNPs–MXene)

Gold nanoparticles (AuNPs) were synthesized by chemical reduction of $AuCl_4^-$ by dissolving Na_3Ctr at 100 °C. First, 100 mL of 1.0 mM $HAuCl_4 \cdot 3H_2O$ containing 200 μL of 1 M NaOH was prepared in a 250 mL round flask. The solution was reacted at 100 °C under stirring, and 10 mL of 38.8 mM Na_3Ctr was rapidly added in a reactor. The reaction was further proceeded for 15 min until changing color, and the synthesized AuNPs were cooled and stored at room temperature.

Ti_3C_2F MXene layers were synthesized via a conventional method. First, 1 g of LiF was dispersed in 7 M HCl solution and stirred continuously for 30 min. Then, 1.5 g of Ti_3AlC_2 MAX was slowly immersed in the solution and continuously stirred at 60 °C for 90 h under ambient conditions. The samples were then separated by centrifugation and re-dispersed in water to obtain a dispersed solution of neutral pH. The samples were finally collected through filtration, followed by drying for 24 h in a vacuum oven at 80 °C.

AuNPs–MXene composites were synthesized by first dissolving 50 mg of Ti_3C_2F MXene layers in 35 mL of distilled water and were subjected to continuous ultrasonication for 3 h. Subsequently, 5 mL of AuNPs was additionally supplemented and ultrasonicated for extra 3 h, in which AuNPs were attached on the surface of MXene. Finally, the samples were

collected by filtration, and the etched samples were dried for 24 h in a vacuum oven at 50 °C.

The morphology and elemental distribution were observed using field-emission transmission electron microscopy (FE-TEM, JEOL, Japan) and high-resolution scanning electron microscopy (HR-SEM, HITACHI, Japan). The crystalline structure of fabricated AuNPs–MXene composites was determined by a multi-purpose X-ray diffractometer (MP-XRD; Pro MRD, RIGAKU, Japan), and the composition in AuNPs-MXene samples was analyzed by Fourier-transform infrared spectroscopy (FT-IR, JASCO, Japan) in a range with 400–4000 cm^{-1}. In order to confirm the chemical composition and the chemical states of nanocomposites, X-ray photoelectron spectroscopy measurements were performed.

Fabrication of peptide on a AuNPs-MXene sensor

The fabrication of AuNPs–MXene and immobilization of peptides on the electrode was done by the following steps. Initially, AuNPs–MXene paste was prepared by sonication AuNPs–MXene suspension (4 mg/mL) containing 0.1 % nafion solution for 1 h [3,4]. Prior to modification, the gold electrode was polished with piranha solution (H_2O_2:H_2SO_4 = 1:3) and washed with distilled water. After that, 15 µL of AuNPs–MXene with 0.1 % nafion was dropped on the polished gold electrode surface and incubated at 25 °C for 3 h. After complete drying, the electrode was immersed in 1 mM MUA overnight and washed with distilled water. The MUA-immobilized electrode was functionalized with 400 mM/100 mM of EDC/NHS and then 100 µg/mL streptavidin solution was added and incubated for 2 h. Then, synthetic peptides were added to functionalized electrode and incubated for 1 h. The peptide-functionalized AuNPs-MXene sensor was incubated in 1 % BSA for 15 min to decrease non-specific binding. Finally, peptide on the AuNPs-MXene sensor was interacted with cathepsin

B and its binding affinity was investigated using SWV.

Table S1. Clinical characteristics of individuals in the detection of cathepsin B

	**Crohns's disease**
Subjects, *n*	8
Gender	
Female	1/8
Male	7/8
Age (years)	12-15
Disease activity	
Mild	3/8
Moderate	3/8
Severe	2/8
Clinical score (CADI)	
Mild	26.7 (average)
Moderate	43.3 (average)
Severe	62.5 (average)

Table S2. The yield of biopanning for cathepsin B

**Round**	**Input (PFU/mL)**	**Output (PFU/mL)**	**Yield (%)**
1st	1.0×10^{11}	2.0×10^{6}	2.0×10^{-5}
2nd	1.0×10^{11}	2.0×10^{7}	2.0×10^{-4}
3rd	1.0×10^{11}	2.0×10^{6}	2.0×10^{-5}
4th	1.0×10^{11}	1.2×10^{7}	1.2×10^{-4}

Table S3. Screening results of biopanning for identifying cathepsin B specific peptides via phage display

**Name**	**Sequence (N→C)**	**Frequency**
CTSB 2-3	DGSMLNRMRGFS	3/78
CTSB 4-1	WDMWPSMDWKAE	8/78
CTSB 4-9	DGFIRPSGVRVA	19/78

**Name**	**Sequence (N→C)**												**Frequency**
CTSB 2-1	D	G	S	M	L	N	R	M	R	G	F	S	3/78
CTSB 4-1	W	D	M	W	P	S	M	D	W	K	A	E	8/78
CTSB 4-9	D	G	F	I	R	P	S	G	V	R	V	A	19/78

Table S4. The synthetic peptides for detection of cathepsin B

Name	Sequence (N→C)	M.W.	Note
CTSB BP1	WDMWPSMDWKAEK - biotin	1935	Selected biopanning and used as scaffold for rational design<br>Incorporated biotin at C-termini for specific immobilization via streptavidin-biotin interaction
CTSB BP2	WGMWPGMGWPAGK - biotin	1686	Designed to see the effect of non-polar residue on binding interaction<br>Incorporated biotin at C-termini for specific immobilization via streptavidin-biotin interaction
CTSB BP3	SSTTNSNSTSNTK - biotin	1554	Designed to see the effect of polar residue on binding interaction<br>Incorporated biotin at C-termini for specific immobilization via streptavidin-biotin interaction
CTSB BP4	HRHRRHRHRKHHK - biotin	2104	Designed to see the effect positive charged residue on binding interaction<br>Incorporated biotin at C-termini for specific immobilization via streptavidin-biotin interaction
CTSB BP5	DDEDDEEDDEDEK - biotin	1822	Designed to see the effect of negative charged residue on binding interaction<br>Incorporated biotin at C-termini for specific immobilization via streptavidin-biotin interaction

Table S5. Analysis of binding site of CTSB BP3 peptide for cathepsin B using molecular docking.

**Peptide**	**Cathepsin B protein**	**Contact distance (Å)**
SER 2	ASN 301	2.664
THR 3	ILE 99	2.738
THR 3	ASN 301	2.744
THR 4	LYS 97	2.540
ASN 5	GLU 98	2.957
ASN 5	ILE 99	2.738
ASN 5	ASN 307	2.537
ASN 5	PHE 309	2.944
ASN 7	ASN 307	3.169
SER 10	ASP 306	2.949
SER 10	LYS 311	2.874
ASN 11	GLU 288	2.536

Table S6. The electroactive surface area of bare gold and AuNPs-MXene fabricated electrodes

**Electrode**	**i_p (A)**	**n**	**$D^{1/2}$ $(cm^2/s)^{1/2}$**	**C $(moles/cm^3)$**	**$v^{1/2}$ $(V/s)^{1/2}$**	**Active surface area (cm^2)**
bare gold electrode	2.61×10^{-4}	1	0.03	2.5×10^{-6}	0.316	0.041
AuNP on gold electrode	2.88×10^{-4}	1	0.03	2.5×10^{-6}	0.316	0.045
MXene on gold electrode	3.18×10^{-4}	1	0.03	2.5×10^{-6}	0.316	0.049
AuNPs-MXene on gold electrode	4.50×10^{-4}	1	0.03	2.5×10^{-6}	0.316	0.071

* Equation: $i_p = (2.69 \times 10^5) \cdot n^{3/2} \cdot A \cdot D^{1/2} \cdot C \cdot v^{1/2}$

Table S7. Detection capability of developed sensors at different concentrations of cathepsin B spiked in human plasma.

Added (nM)	Found (nM)	CV (%)	Recovery (%)
**Phage-on-a sensor**			
31.3	28.4 ± 1.1	3.85	91.05 ± 3.51
62.5	56.7 ± 4.6	8.13	90.67 ± 7.37
125	116.9 ± 10.4	8.89	93.58 ± 8.33
**Peptide-on-a sensor**			
31.3	30.1 ± 2.1	6.99	96.03 ± 6.72
62.5	61.5 ± 1.5	2.37	98.37 ± 2.33
125	124.3 ± 1.6	1.30	99.43 ± 1.29
**Peptide-on-a-AuNPs/MXene sensor**			
31.3	29.5 ± 0.5	1.68	94.52 ± 1.58
62.5	62.4 ± 2.6	4.14	99.84 ± 4.13
125	126.4 ± 7.6	6.04	101.16 ± 6.11

Table S8. Comparison of the analytical performance for the detection of cathepsin B

Detection method	Target	Receptor	Recovery percentage (%)	Ref
Digital ELISA	Cathepsin B	Antibody	70 - 130	[5]
SPRi biosensor	Cathepsin S	Antibody	98 - 101	[6]
Carbon nanofiber nanoelectrode array	Cathepsin B	Peptide as substrate	Not described	[7]
Nanoporous alumina-based optical sensor	Cathepsin B	Human serum albumin -thionine	94	[8]
Activity based-electrochemical sensor	Cathepsin B	Peptide as substrate	140 ± 20	[9]
Differential pulse voltammetry-based sensor	Cathepsin L	Cystatin C	Not described	[10]
Electrochemical peptide-AuNPs/Mxene based sensor	Cathepsin B	Affinity peptide	94.52 - 101.16	This study

Table S9. Analytical results between our developed sensor and the reference ELISA with Crohn's patient samples.

**Patient group**		**ELISA (nM)**	**Peptide-on-a-AuNPs/MXene sensor (nM)**	**Recovery (%)**	**RSD (%)**
Mild	S1	3.89 ± 0.47	3.89 ± 0.36	100.23 ± 6.87	6.85
	S2	2.27 ± 0.40	1.94 ± 0.22	86.13 ± 5.20	6.03
	S3	0.69 ± 0.19	0.67 ± 0.16	99.51 ± 11.92	11.98
Moderate	S4	16.21 ± 1.42	16.48 ± 1.68	101.72 ± 5.83	5.74
	S5	7.39 ± 0.93	7.37 ± 0.87	99.73 ± 0.89	0.90
	S6	0.43 ± 0.32	0.44 ± 0.32	102.85 ± 8.47	8.24
Severe	S7	32.58 ± 2.02	33.53 ± 2.69	102.83 ± 1.96	1.91
	S8	22.54 ± 2.19	21.83 ± 2.04	97.19 ± 9.59	9.86

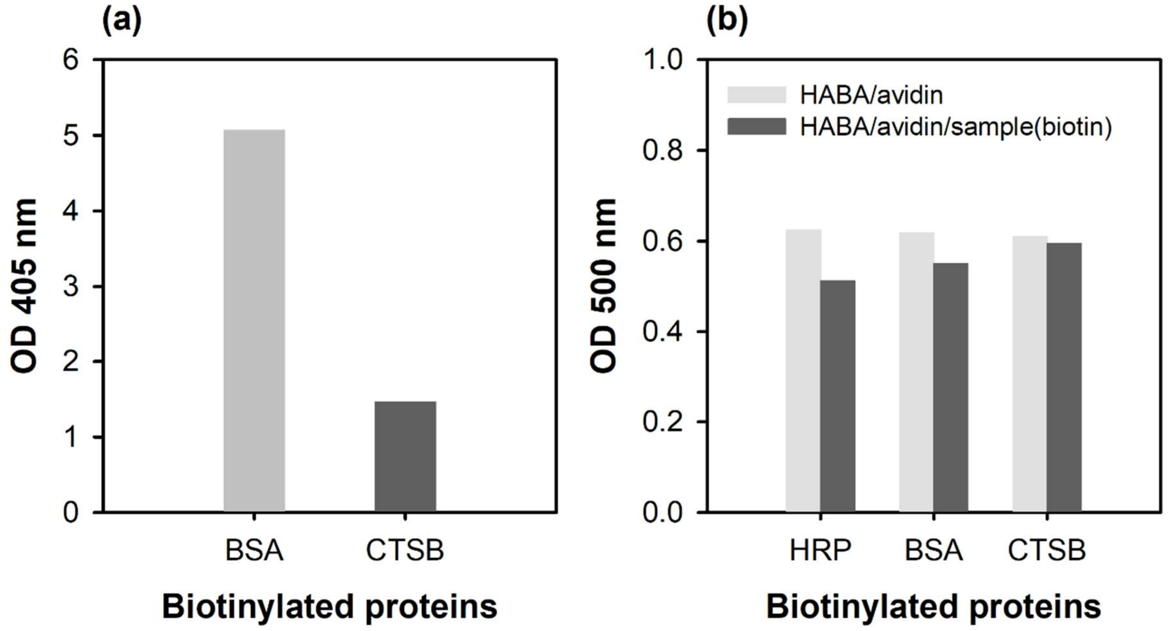

(c)

Biotinylated protein	A_{500} HABA/avidin	A_{500} HABA/avidin/biotin	Concentration (mg/mL)	Biotin ratio (biotin/mole of protein)
HRP (control)	0.625	0.513	2.5	1.054
BSA (control)	0.619	0.551	0.859	3.073
CTSB	0.61	0.595	0.065	3.745

* Molecular weight: HRP (40kDa), BSA (66 kDa), CTSB (27.5 kDa)

Figure S1. The validation assay for biotinylated cathepsin B protein. (a) The biotin-labelled BSA (250 nM = 16.5 μg/mL) and CTSB (250 nM = 6.875 μg/mL) were immobilized on streptavidin coated plate, and it was confirmed using HRP conjugated streptavidin and ABTS at 405 nm. (b) To quantitate labelled biotin, the biotinylated cathepsin B was added to the mixture of HABA and avidin and biotinylated HRP and BSA were used as control. (c) Biotin ratio was calculated from biotin quantitation results, which biotin ratio for cathepsin B was about 3.745.

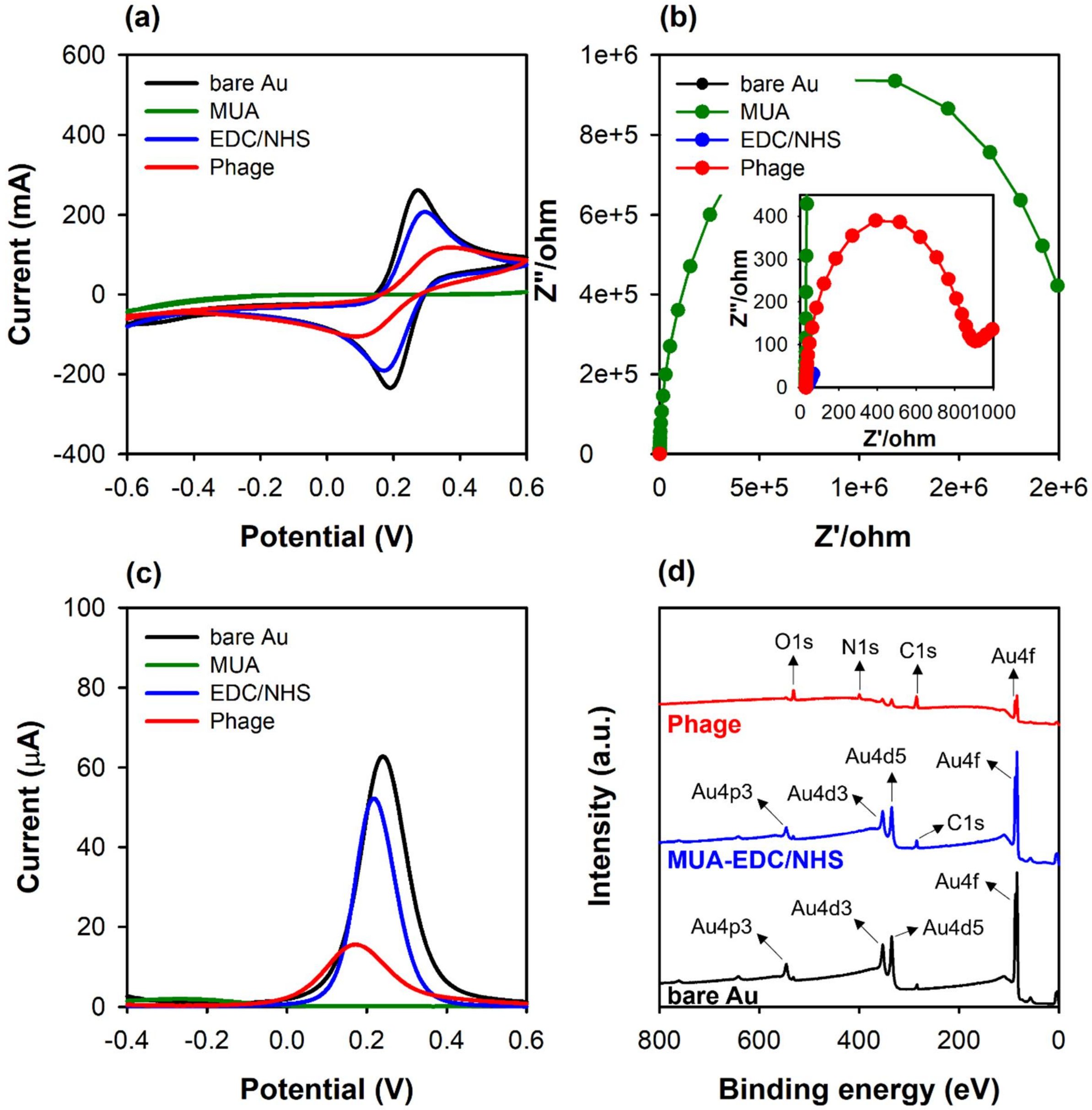

Figure S2. The responses of electrochemical measurements of phage-on-a sensor. (a-c) CV, EIS, and SWV responses for preparation steps of phage sensor (bare Au, Au@1mM MUA, Au@ 400mM/100mM EDC/NHS, Au@4-1 phage). It was conducted at 1 M KNO_3 with 2.5 mM $[Fe(CN)_6]^{3-/4-}$. (d) X-ray photoelectron spectroscopy (XPS) spectra of bare Au, Au@MUA-EDC/NHS, Au@4-1 phage.

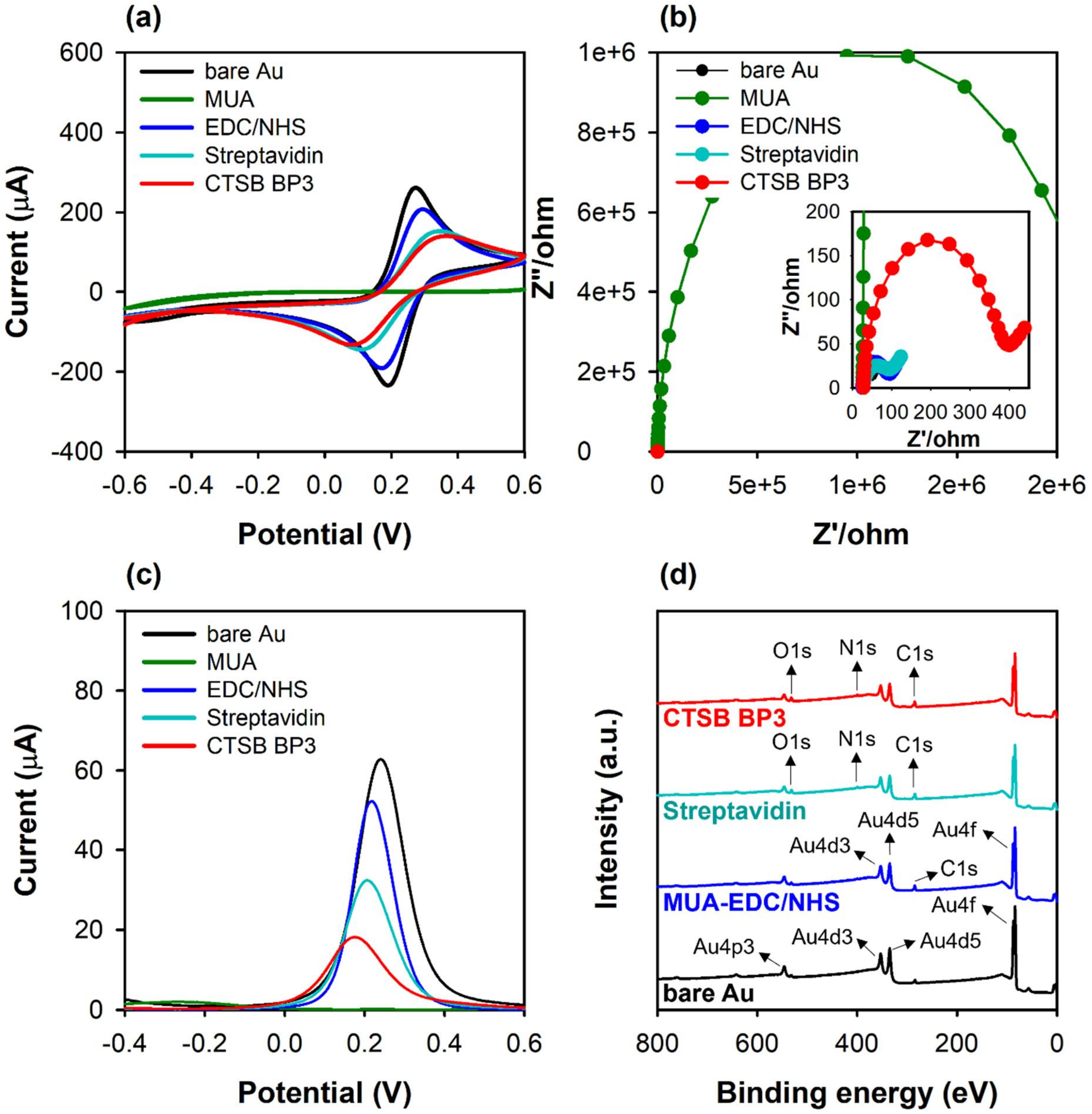

Figure S3. The responses of electrochemical measurements of peptide-on-a sensor. (a-c) CV, EIS, and SWV responses for preparation steps of phage sensor (bare Au, Au@1 mM MUA, Au@ 400 mM/100 mM EDC/NHS, Au@streptavidin, Au@CTSB BP3 peptide). It was conducted at 1 M KNO_3 with 2.5 mM $[Fe(CN)_6]^{3-/4-}$. (d) X-ray photoelectron spectroscopy (XPS) spectra of bare Au, Au@MUA-EDC/NHS, Au@streptavidin, Au@CTSB BP3 peptide.

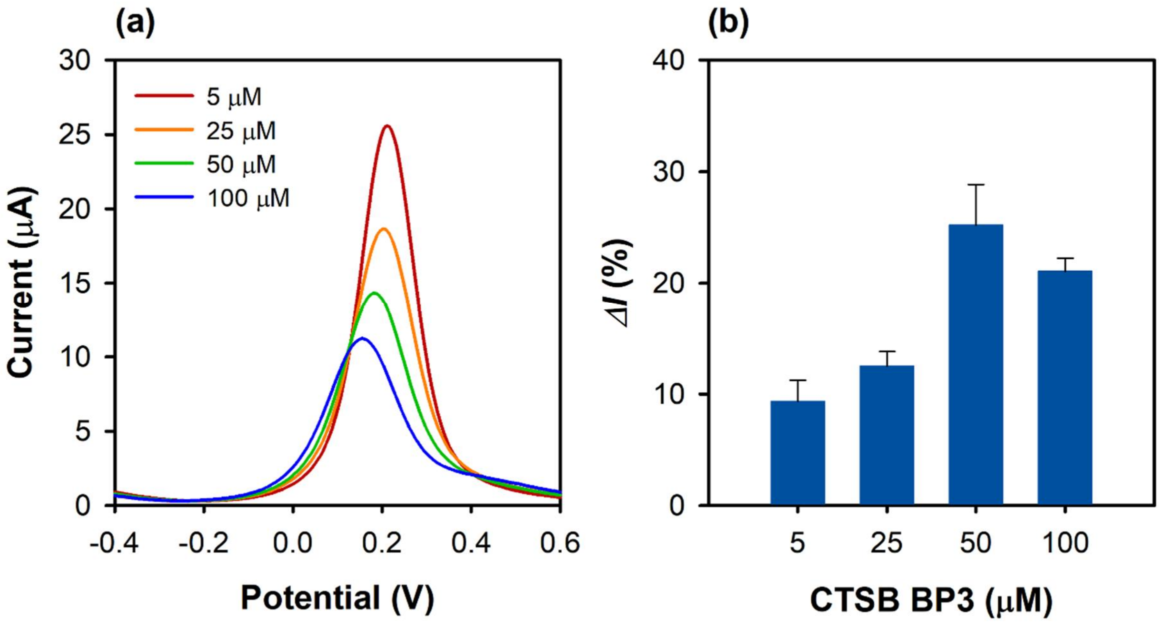

Figure S4. Effect of peptide concentration for peptide-on-a sensor. (a) The current responses of different CTSB BP3 peptide concentrations (5 – 100 μM). (b) The current changes of peptide-on-a sensor in different CTSB BP3 peptide concentrations for cathepsin B.

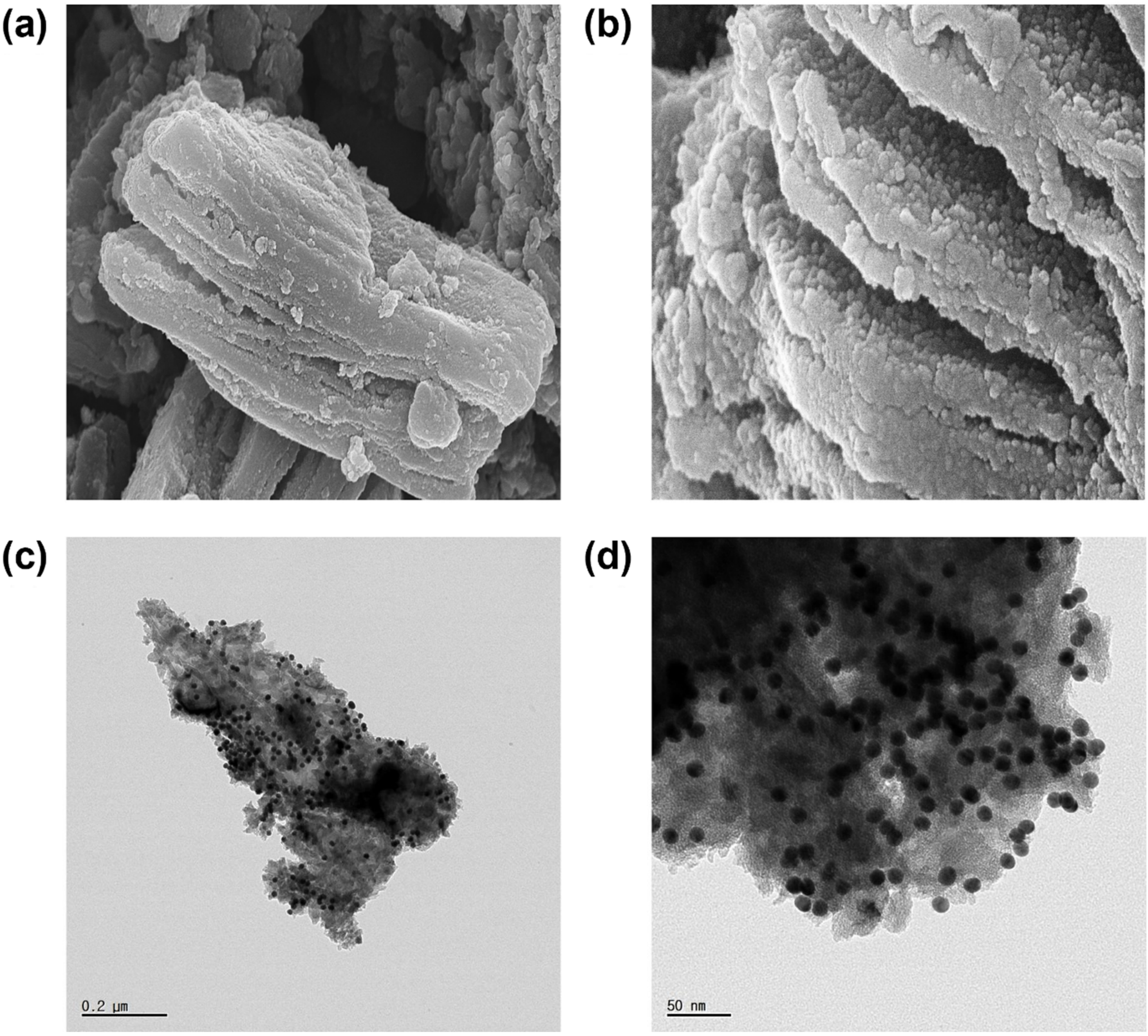

Figure S5. Morphological characterization of AuNPs-MXene composite; SEM images under (a) low and (b) high resolution; TEM images under (c) low and (d) high resolution.

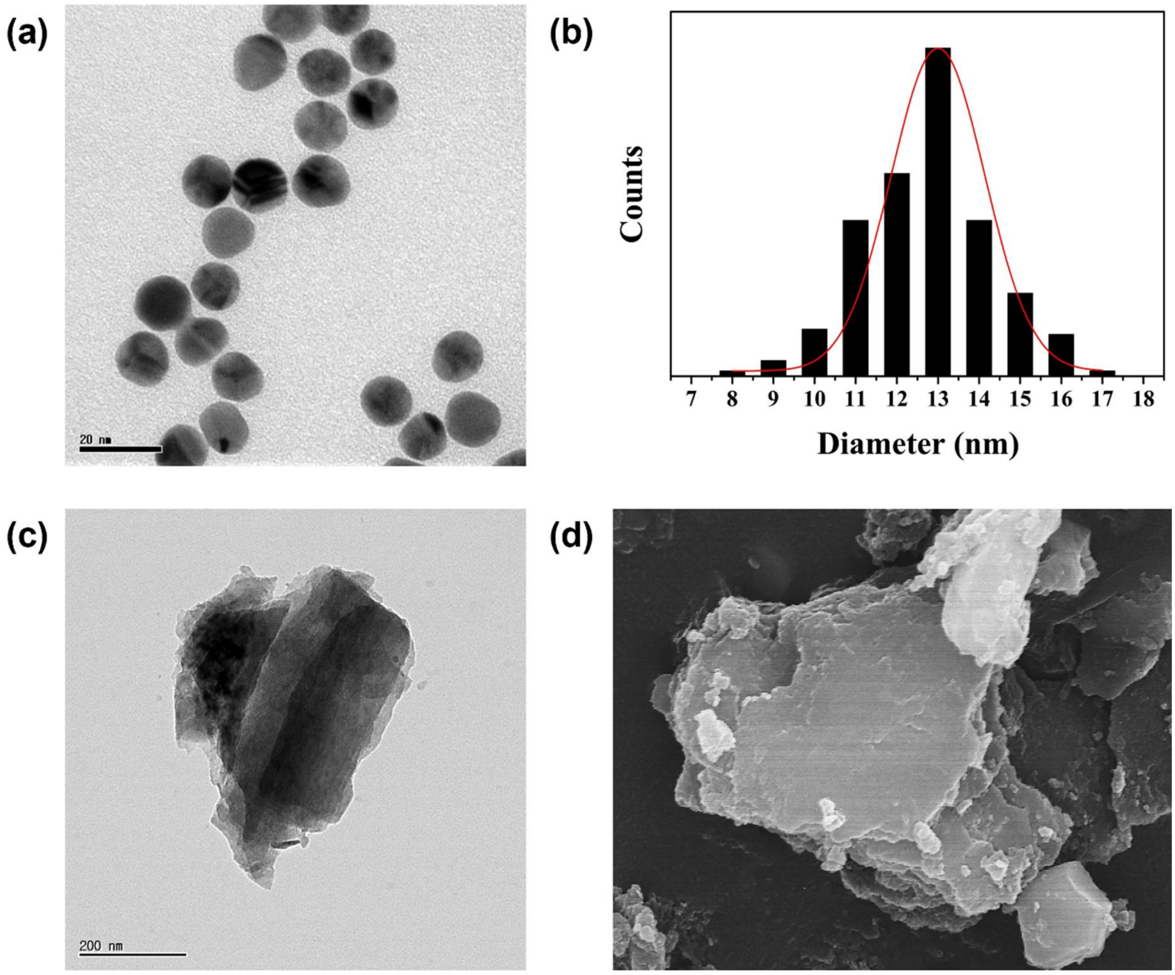

Figure S6. (a) TEM image and (b) size distribution graph of pre-synthesized Au NPs. (c) TEM image and (d) SEM image of Ti_3C_2F MXene.

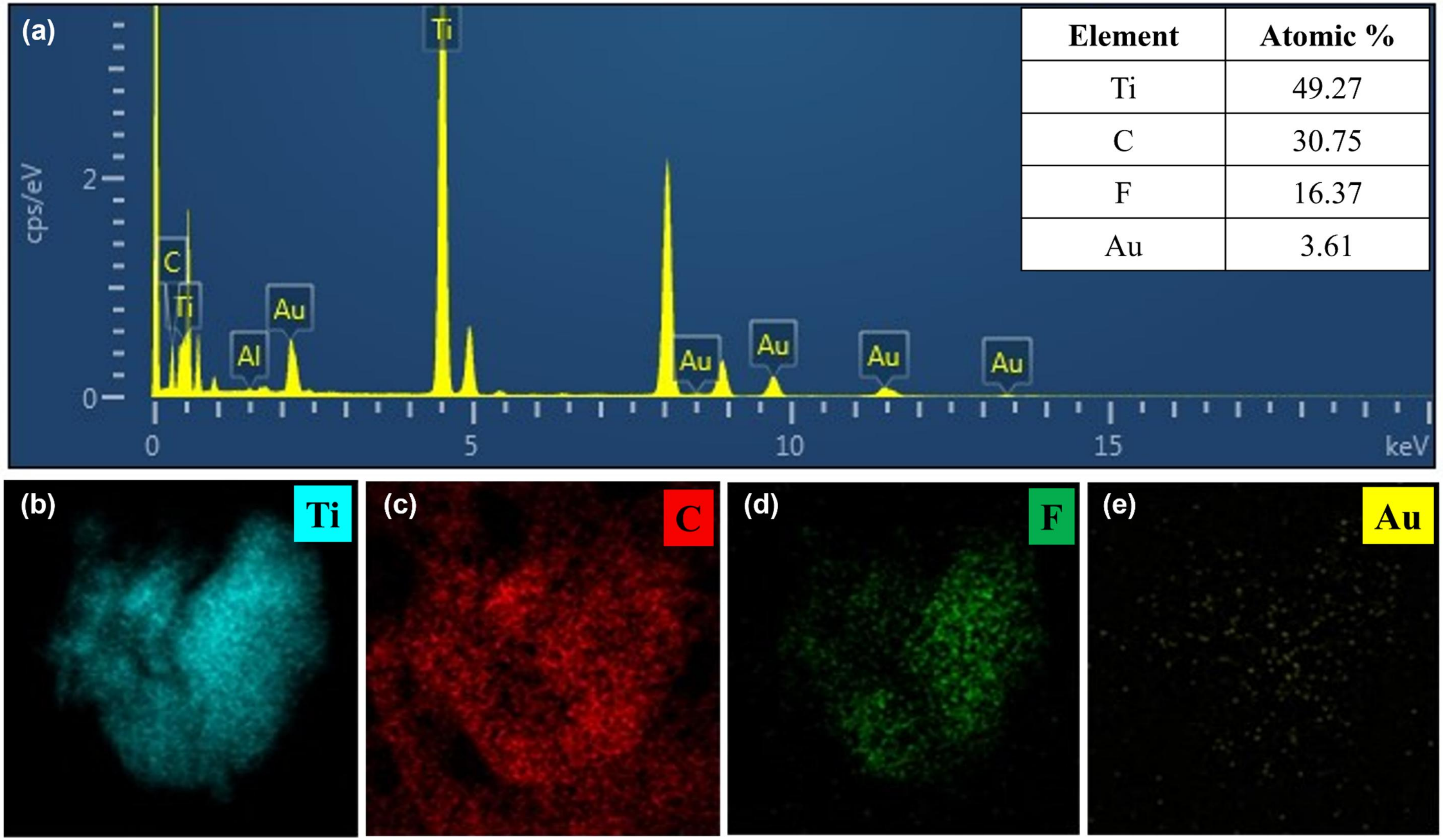

Element	Atomic %
Ti	49.27
C	30.75
F	16.37
Au	3.61

Figure S7. (a) EDS spectrum of AuNPs-MXene composites. (b-e) Elemental mapping images obtained from EDS for elements Ti, C, F and Au, respectively.

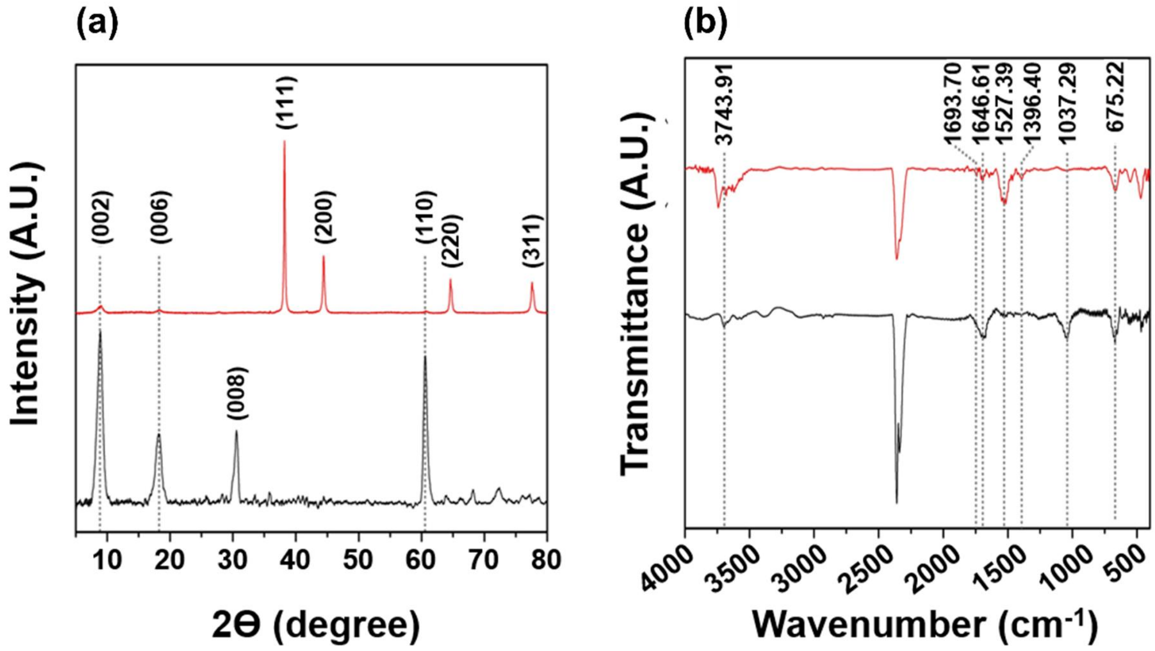

Figure S8. (a) X-ray diffraction spectra and (b) FT-IR spectra of AuNPs-MXene composites (red line) and MXene (black line).

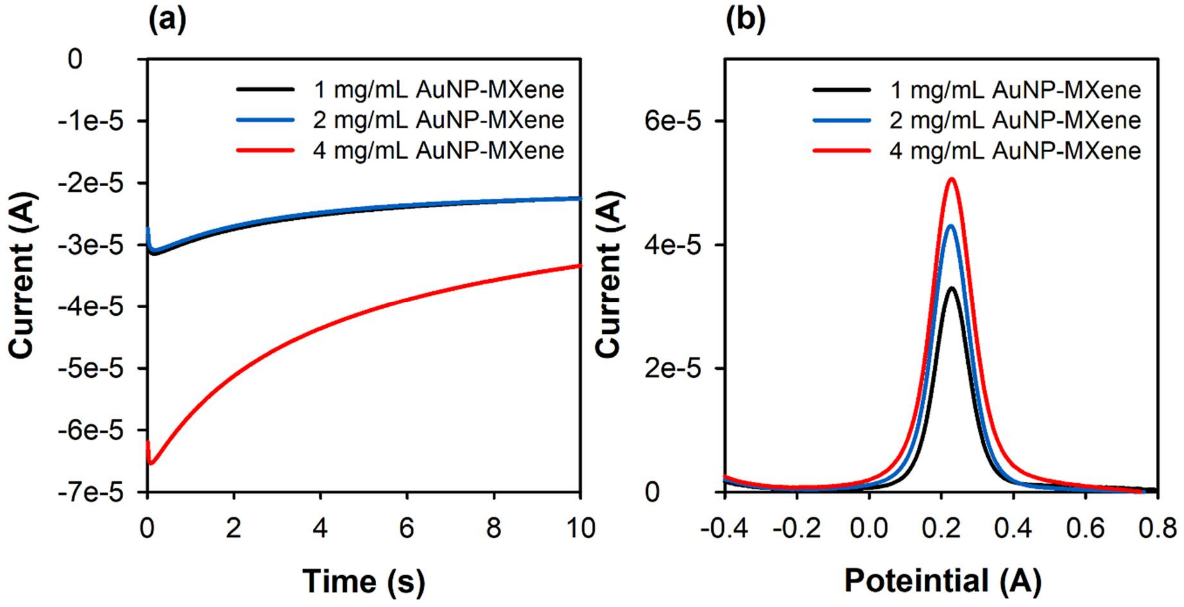

Figure S9. Optimization of AuNPs-MXene concentration and comparison of the effect of scan rate on gold electrode. (a) Chronoamperometric response of the sensor with different concentration of AuNPs-MXene. (b) Square wave voltammetry analysis of the sensor with different concentration of AuNPs-Mxene.

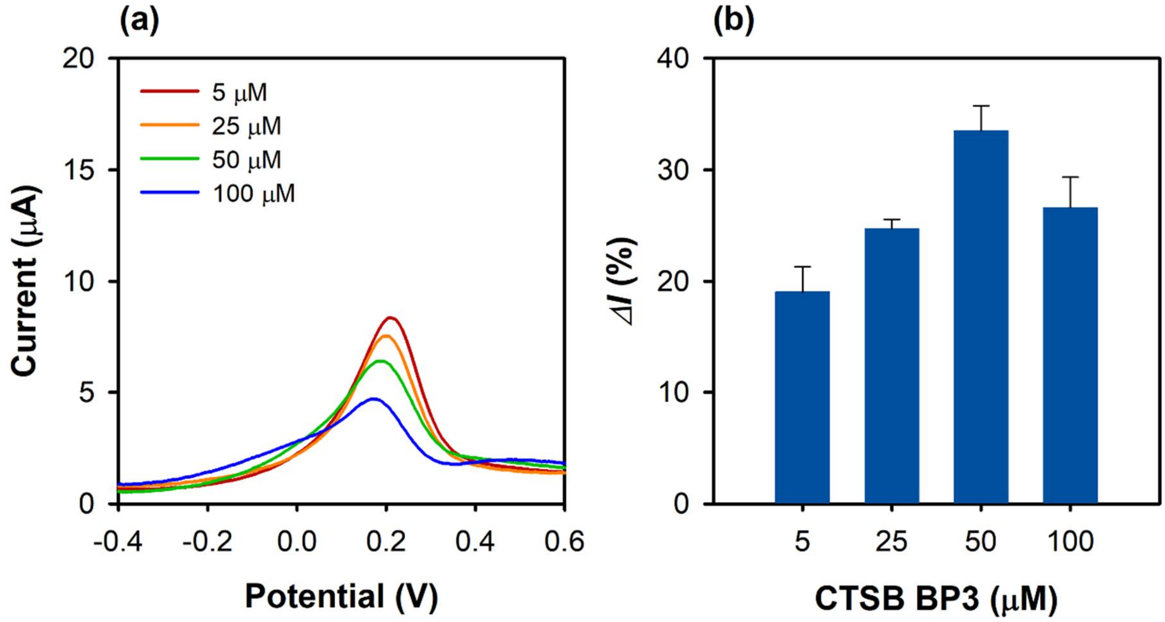

Figure S10. Effect of peptide concentration for peptide-on-a-AuNPs/MXene sensor. (a) The current responses of different CTSB BP3 peptide concentrations (5 – 100 μM). (b) The current changes of peptide-on-a-AuNPs/MXene sensor in different CTSB BP3 peptide concentrations for cathepsin B.

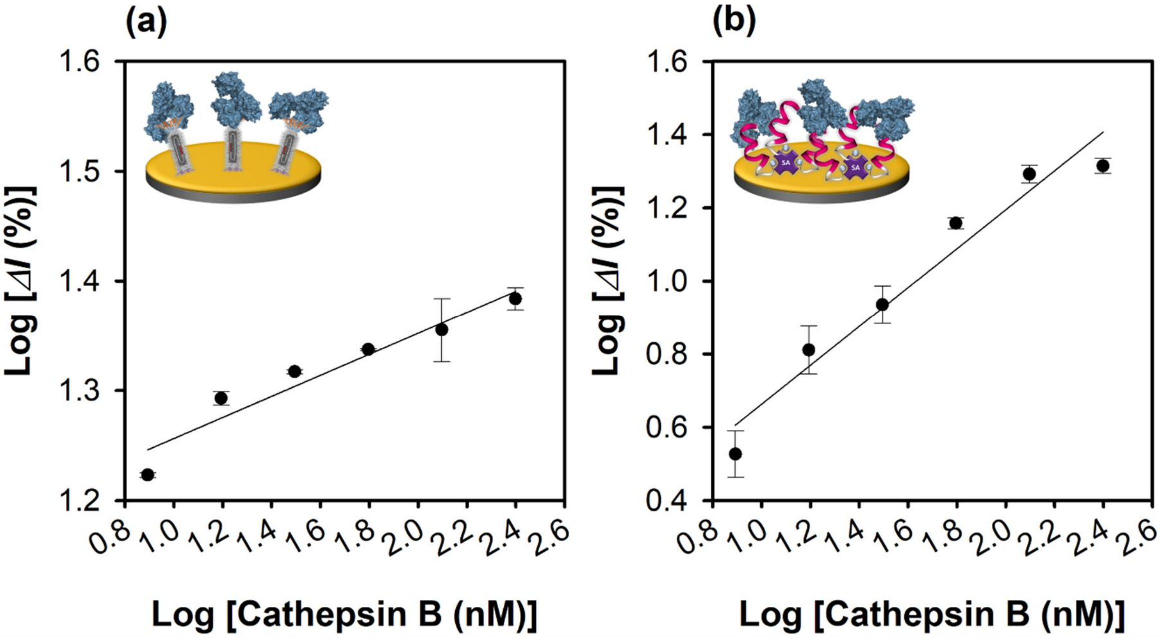

Figure S11. Comparison of standard curves of the developed sensors: a) Phage-on-a-sensor, b) Peptide-on-a-sensor. The change of current was measured by SWV with 1 M KNO_3 containing 2.5 mM $[Fe(CN)_6]^{3-/4-}$. In phage-on-a-sensor, the titer of phages was 1×10^{12} PFU/mL, while the concentration of peptide used in peptide-on-a-sensor was 50 μM. All measurements were done in triplicate, and error bars represent standard deviations.

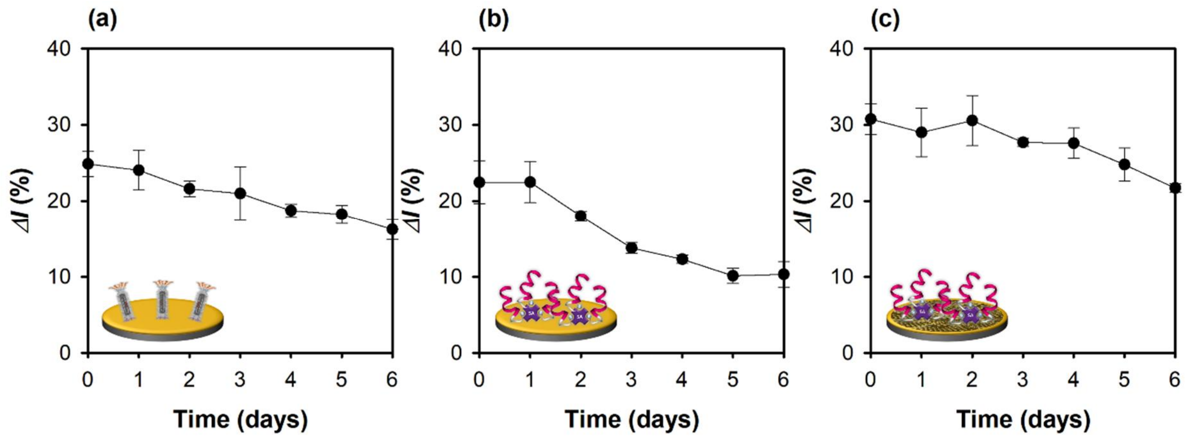

Figure S12. Comparison of stability of the developed sensor. (a) Phage-on-a sensor, (b) peptide-on-a sensor, (c) peptide-on-a-AuNPs/MXene sensor.

Additional References

[1] Xue Y.R., Li X., Li H.B., Zhang W.K., Quantifying thiol-gold interactions towards the efficient strength control, Nat. Commun. 2014; 5 : 4348.

[2] Karoonuthaisiri N., Charlermroj R., Morton M.J., Oplatowska-Stachowiak M., Grant I.R., Elliott C.T., Development of a M13 bacteriophage-based SPR detection using Salmonella as a case study, Sens. Actuators B Chem. 2014; 190: 214-220.

[3] Kumar S., Lei Y., Alshareef N.H., Quevedo-Lopez M., Salama K.N., Biofunctionalized two-dimensional Ti3C2 MXenes for ultrasensitive detection of cancer biomarker, Biosens. Bioelectron. 2018; 121: 243-249.

[4] Rasheed P.A., Pandey R.P., Rasool K., Mahmoud K.A., Ultra-sensitive electrocatalytic detection of bromate in drinking water based on Nafion/Ti3C2Tx (MXene) modified glassy carbon electrode, Sens. Actuators B Chem. 2018; 265: 652-659.

[5] Thangavelu B., Boutté A.M., Single Molecule Assay for Ultrasensitive Detection of Cathepsin B in Human Blood, ACS Omega 2021; 6: 9609-9616.

[6] Oldak L., Sankiewicz A., Żelazowska-Rutkowska B., Cylwik B., Lukaszewski Z., Skoczylas M., Gorodkiewicz E., Two SPRi biosensors for the determination of cathepsin S in blood plasma, Talanta 2021; 225: 121900.

[7] Song Y., Fan H., Anderson M.J., Wright J.G., Hua D.H., Koehne J., Meyyappan M., Li J., Electrochemical activity assay for protease analysis using carbon nanofiber nanoelectrode arrays, Anal. Chem. 2019; 91: 3971-3979.

[8] Tabrizi M.A., Ferré-Borrull J., Marsal L.F., An optical biosensor for the determination of cathepsin B as a cancer-associated enzyme using nanoporous anodic alumina modified with human serum albumin-thionine, Microchim. Acta 2020; 187: 1-9.

[9] Song Y., Wright J.G., Anderson M.J., Rajendran S., Ren Z., Hua D.H., Koehne J.E., Meyyappan M., Li J., Quantitative Detection of Cathepsin B Activity in Neutral pH Buffers Using Gold Microelectrode Arrays: Toward Direct Multiplex Analyses of Extracellular Proteases in Human Serum, ACS Sens. 2021; 6: 3621-3631.

[10] Ranade H., Paliwal P., Chaudhary A.A., Piplani S., Rudayni H.A., Al-Zharani M., Niraj R.R., Datta M., Predicting Diagnostic Potential of Cathepsin in Epithelial Ovarian Cancer: A Design Validated by Computational, Biophysical and Electrochemical Data, Biomolecules 2022; 12: 53.
